# Accelerated Electro‐Conversion of a Nickel Coordination Complex for Hybrid Water Electrolysis

**DOI:** 10.1002/smll.202507907

**Published:** 2025-08-29

**Authors:** Nikhil N. Rao, Avani Anil Kumar, Peter Kúš, Chandraraj Alex, Muhammed Safeer Naduvil Kovilakath, Tomáš Hrbek, Iva Matolínová, Neena S. John

**Affiliations:** ^1^ Centre for Nano and Soft Matter Sciences, Shivanapura Bengaluru 562162 India; ^2^ Manipal Academy of Higher Education Manipal 576104 India; ^3^ Faculty of Mathematics and Physics Department of Surface and Plasma Science Charles University V Holešovičkách 2 Prague 8 180 00 Czech Republic; ^4^ Institute of Inorganic Chemistry Kiel University Otto‐Hahn‐Platz 10 24118 Kiel Germany

**Keywords:** activation, coordination complex, nickel, pre‐catalyst, transformation, urea electro‐oxidation

## Abstract

Electrocatalytic energy conversion relies on the dynamic transformation of electrode materials into “electrocatalytically active phases” under reaction conditions. Pre‐catalysts, which undergo extensive structural and chemical changes during electrochemical activation, are particularly promising in this regard. In the context of electrocatalysis, coordination complexes with labile ligands offer a unique advantage, as they can rapidly reconstruct under electrochemical conditions. Herein, a hydrazine‐coordinated Ni complex embedded in a conductive carbon nanotube matrix is presented as a pre‐catalyst for urea‐assisted hybrid water electrolysis, that transforms into highly active γ‐NiOOH nanosheets on electrochemical activation, demonstrating exceptional urea electrooxidation performance, with a low Tafel slope of 21.6 mV dec^−1^, a high turnover frequency (TOF) of 0.0728 s^−1^, and stable operation over 40 h of continuous electrolysis, reflecting superior catalytic kinetics and excellent durability. In situ synchrotron X‐ray absorption, Raman, and electrochemical impedance spectroscopy reveal the dynamic evolution of active sites, the underlying reaction mechanism, and the fate of the active species after prolonged electrolysis. The integration of this pre‐catalyst into an anion‐exchange membrane electrolyzer highlights its potential for practical application. This work showcases the transformative role of Ni‐based coordination complexes as pre‐catalysts, offering an innovative blueprint for the rational design of high‐performance urea oxidation electrocatalysts.

## Graphical abstract

A nickel coordination complex pre‐catalyst undergoes rapid electrochemical activation to form highly active nanosheets that enable efficient and stable urea electrolysis. Structural evolution and the operating mechanism are revealed through in situ spectroscopy, and practical viability is demonstrated in an electrolyzer. This smart (pre‐)catalyst design strategy, enables cleaner and more efficient hydrogen generation.

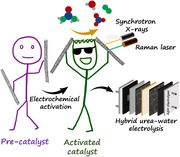

## 1 Introduction

The urea electro‐oxidation reaction (UOR) has attracted significant attention for its potential to lower the energy required for hydrogen production in hybrid water electrolysis.^[^ ^1^ ^]^ Among various transition metal‐based electrocatalysts, Ni‐based materials stand out due to their excellent UOR activity, attributed to the formation of nickel oxyhydroxide (NiOOH) species under anodic conditions.^[^ ^2^ ^]^ NiOOH exhibits optimal adsorption energies for urea and its reaction intermediates, thereby serving as the “active species” for UOR.^[^ ^3^ ^]^ However, due to the instability of these species under atmospheric conditions, their direct chemical synthesis and utilization are challenging, thereby necessitating the in situ formation of these species under electrochemical conditions.^[^ ^4^ ^]^ Consequently, there is a strong demand for Ni‐based materials that electrochemically transform into highly active catalytic phases with an abundant number of NiOOH active sites with high electrocatalytic turnover frequency and stability toward long‐term electrolysis. In this regard, Ni‐based pre‐catalysts emerge as ideal candidates. Pre‐catalysts refer to materials that undergo electrochemical transformation before becoming the actual electrocatalyst.^[^ ^5^ ^]^ In fact, most Ni‐based UOR catalysts function as pre‐catalysts, as they undergo structural changes during electrochemical activation.^[^ ^6^ ^]^ However, the extent of catalyst reconstruction varies across different pre‐catalysts and could depend on various factors such as crystallinity, microstructure, and the presence of unstable moieties such as active metals, ligands, or non‐metal species.^[^ ^5^ ^]^ The chemical processes that drive the reconstruction of the pre‐catalyst include redox reactions, hydrolysis, combination, dissociation, or double decomposition, which lead to changes in the valence state of the metal centers, leaching, redeposition, and/or random atom migration.^[^ ^5^, ^7^ ^]^ Clearly, enhancing the degree of constructive electrochemical transformation of the pre‐catalyst could significantly enhance the electrocatalytic activity. One suitable approach to achieve this is to exploit the inherent instability of coordination complexes or metal‐organic frameworks in different media to accelerate their conversion into desired active species under operating electrochemical conditions.^[^ ^8^ ^]^ Several transition metal coordination complexes, salts, metal‐organic frameworks, etc., have been employed as pre‐catalysts for a wide range of electrocatalysed reactions. Coordination complex pre‐catalysts for the oxygen evolution reaction (OER)‐Fe‐doped nickel hydrogen cyanamide,^[^ ^9^ ^]^ dithiocarbamate and diphosphine coordination complexes of Ni,^[^ ^10^, ^11^ ^]^ Ni‐Co Prussian blue analogues,^[^ ^12^ ^]^ Fe phthalocyanine tetrasulfonate on nickel foam,^[^ ^13^ ^]^ NiFe‐tannin complex film,^[^ ^14^ ^]^ and bis(germylene)‐Ni complex^[^ ^15^ ^]^ have been shown to generate Ni(Fe)O_x_H_y_‐like species under electrochemical conditions. Metal‐organic frameworks (MOFs) constitute another class of pre‐catalysts that electrochemically transform into the active phase for OER and UOR.^[^ ^16^, ^17^, ^18^, ^19^, ^20^, ^21^, ^22^ ^]^ Intriguingly, even soluble salts of metals have been demonstrated to behave as pre‐catalysts for electrocatalyzed reactions.^[^ ^23^, ^24^ ^]^ Most of the above‐mentioned pre‐catalysts have been primarily explored for their OER activity, with limited studies on UOR—a comparatively more complex reaction involving a delicate balance between active site evolution, utilization, and regeneration. Though the active species formed from the above‐mentioned pre‐catalysts are identified, in situ spectro‐electrochemical studies on the dynamic evolution of these species during electrocatalytic UOR are lacking. Importantly, these studies have rarely demonstrated the practical implementation of such pre‐catalysts in alkaline electrolyzers, leaving a critical gap between fundamental understanding and device‐level application.

In order to gain an in‐depth understanding of the importance of active sites that are in situ generated from the electrochemical activation of pre‐catalysts, we have synthesized a Ni‐based coordination complex—nickel hydrazine chloride—as a pre‐catalyst for UOR, whose electrical conductivity and, therefore, the charge transfer are enhanced by enmeshing it in a functionalized multiwalled carbon nanotube (CNT) matrix. Using several characterization techniques, the chemical nature of the active phase formed after the electrochemical activation of this pre‐catalyst is investigated. Furthermore, the high UOR activity and excellent stability of the activated catalyst are demonstrated by three‐electrode, two‐electrode, and anion exchange membrane (AEM) electrolyzer measurements. The activated catalyst demonstrates a current density of 100 mA cm^−2^ at 1.50 V versus RHE, a 230 mV reduction in potential compared to OER to reach 100 mA/cm^2^, a low Tafel slope of 21.6 mV dec^−1^, and a high turnover frequency (TOF) of 0.0728 s^−1^, indicating fast reaction kinetics and high intrinsic efficiency of the active sites. In situ Raman, X‐ray absorption spectroscopy (XAS), and electrochemical impedance spectroscopy (EIS) are performed to study the dynamic changes in the active species formed in this system and ascertain the operating reaction mechanism. With this work, we aim to address the following scientific questions: a) Can a Ni coordination complex serve as an efficient pre‐catalyst for UOR? b) What structural and compositional transformations does the pre‐catalyst undergo during electrochemical activation? c) What is the dynamic nature and ultimate fate of the active species formed? d) Can this pre‐catalyst be effectively integrated into electrolyzers?

## 2 Results

### 2.1 Pre‐catalyst Characterization

The powder X‐ray diffraction (XRD) pattern of the as‐synthesized nickel hydrazine chloride (NiHyd) complex obtained by the simple solution route of synthesis show multiple peaks that agree with that of Ni(N_2_H_4_)_3_Cl_2_ given in earlier reports (Figure S1a, Supporting Information).^[^ ^25^, ^26^ ^]^ From a combination of inductively coupled plasma optical emission spectroscopy (ICP‐OES), elemental analyzer, and silver nitrate‐precipitation titrimetry, the NiHyd complex is found to be composed of 29.4% (±0.1) of Ni, 35.5% (±0.1) of Cl, 32.15% (±0.13) of N and 5.05% (±0.12) of H. The Fourier transform infrared (FTIR) spectra (Figure S1b, Supporting Information) has revealed details about the types of bonds/functional groups present in NiHyd@CNT‐PC (abbreviation for NiHyd on multiwalled carbon nanotubes‐pre‐catalyst) as listed in Table S1 (Supporting Information), which are also in accordance with the FTIR profile of [Ni(N_2_H_4_)_3_]Cl_2_ reported previously.^[^ ^25^ ^]^ Field emission electron microscopy (FESEM) micrographs of NiHyd@CNT‐PC clearly show the incorporation of the complex in a CNT matrix when compared to that of the pristine complex (NiHyd) (Figure S1c,d, Supporting Information), transmission electron microscopy (TEM) micrograph of NiHyd@CNT‐PC further corroborates the growth of NiHyd complex on CNT providing intimate contact between the complex and CNT, beneficial for enhancing the electrical conductivity and therefore charge transfer characteristics of the pre‐catalyst, and ultimately, the catalyst for UOR (Figure S1e, Supporting Information).

### 2.2 Electrochemical Activation and UOR Performance

**Figure 1:**
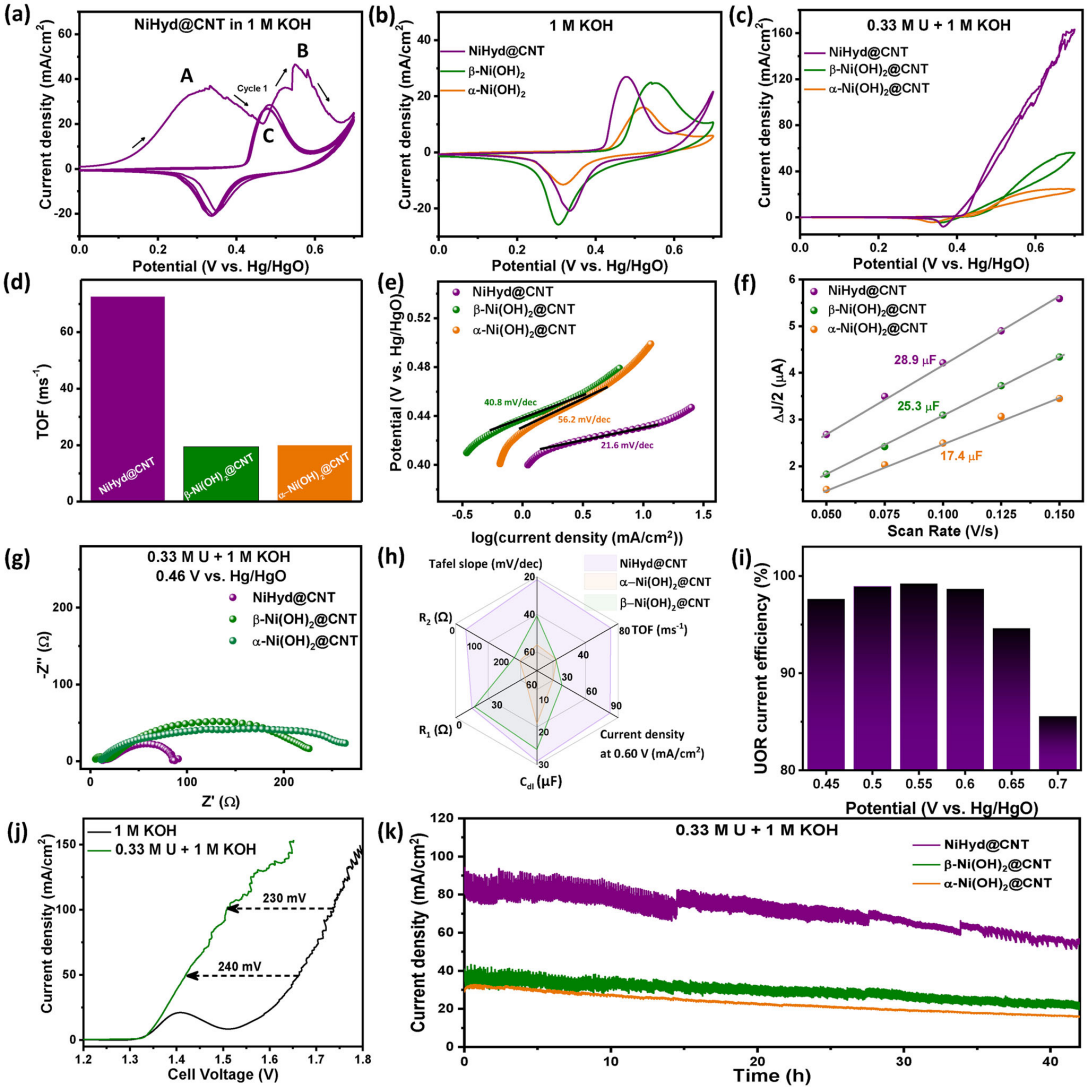
CVs of a) Activation of NiHyd@CNT in 1 m KOH, b) Activated NiHyd@CNT, β‐Ni(OH)_2_@CNT, and α‐Ni(OH)_2_@CNT in 1 m KOH c) 0.33 m urea + 1 m KOH; d) Comparison of TOF values calculated at 0.60 V versus Hg/HgO; e) Tafel plot; f) Calculation of C_dl_ values; g) Nyquist plots recorded in 0.33 m urea + 1 m KOH; h) Spider plot to compare the figures of merit; i) UOR current efficiency of NiHyd@CNT from step‐voltage experiment; j) LSVs recorded in two‐electrode configuration to compare urea‐assisted water splitting and conventional water splitting in the two‐electrode configuration and k) i‐t curve of all the catalysts in 0.33 m urea + 1 m KOH.

The NiHyd@CNT‐PC is subjected to 10 cycles of cyclic voltammetry (CV) from 0 to 0.7 V versus Hg/HgO in 1 m KOH at a scan rate of 10 mV s^−1^, which constitutes the electrochemical activation step (**Figure** **1**a). The first activation CV shows two peaks – one before the redox region of Ni^2+^/Ni^3+^ (peak A, Figure 1a), which could correspond to the oxidation of hydrazine ligands, and one at the Ni^2+^/Ni^3+^ redox region (peak B, ≈0.42 V versus Hg/HgO). After the first cycle, the first peak disappears, and only the Ni^2+^/Ni^3+^ redox peaks become prominent (peak C). This suggests the complete oxidation of hydrazine ligands during the first scan, indicating complete transformation of the coordination complex. To confirm the origin of the first peak, the Ni‐felt electrode is electrochemically activated in 1 m KOH, and voltammograms are recorded in 0.1 m N_2_H_4_.H_2_O in 1 m KOH (Figure S2a, Supporting Information). It w observed that the hydrazine oxidation reaction occurred at a much lower potential than the usual Ni^2+^/Ni^3+^ redox region, thereby attributing the first peak (peak A) in the first cycle of electrochemical activation of NiHyd@CNT‐PC to electrochemical oxidation of the hydrazine ligands.

The UOR activity parameters of activated NiHyd@CNT (hereafter referred to as NiHyd@CNT) are compared to those of both α and β‐Ni(OH)_2_, which are widely employed UOR electrocatalysts that furnish NiOOH species on electrochemical activation.^[^ ^27^, ^28^, ^29^, ^30^, ^31^, ^32^, ^33^, ^34^, ^35^, ^36^ ^]^ To nullify the influence of the conducting additive, the α and β‐Ni(OH)_2_ catalysts are also supplemented with CNT during electrode preparation and subjected to the same activation protocol (Figure S2b,c, Supporting Information). The CVs of NiHyd@CNT, α‐Ni(OH)_2_@CNT, and β‐Ni(OH)_2_@CNT recorded in 1 m KOH are shown in Figure 1b. The anodic peak that onsets at ≈0.42 V versus Hg/HgO represents the oxidation of Ni^2+^ to Ni^3+^, while the cathodic peak in the reverse scan originates from the reduction of Ni^3+^ to Ni^2+^. When the CVs are recorded in an electrolyte containing 0.33 m urea + 1 m KOH, there is a drastic rise in the anodic current density, suggesting the occurrence of UOR (Figure 1c and Figure S2d, Supporting Information). Figure 1c highlights the dramatically high UOR current density of NiHyd@CNT when compared to α‐Ni(OH)_2_@CNT, and β‐Ni(OH)_2_@CNT.

In order to optimize the kind of C‐additive for optimum UOR activity, NiHyd complex incorporated in reduced graphene oxide (rGO), Vulcan carbon (VC), and CNT are compared for their UOR activity (Figure S2e, Supporting Information). While CNT emerged as the most effective carbon additive, its optimal amount during synthesis was also determined (Figure S2f, Supporting Information). Furthermore, the loading amount of the catalyst is also an important factor for determining the subsequent electrocatalytic activity parameters.^[^ ^37^ ^]^ It was observed that a catalyst loading of 1.14 mg cm^−2^ yielded the highest UOR current density (Figure S2g, Supporting Information). Furthermore, the UOR activity of NiHyd@CNT also outperforms that of chemically synthesized NiOOH@CNT, as shown in Figure S2h (Supporting Information). The electrochemical activation protocol for NiHyd@CNT‐PC has been optimized by varying the potential window and scan rate, with the best UOR performance obtained at a 0–0.70 V range and a scan rate of 10 mV s^−1^ (Figure S3, Supporting Information).^[^ ^38^ ^]^

NiHyd@CNT exhibits a substantially higher TOF of 0.0728 s^−1^ compared to α‐ and β‐Ni(OH)_2_@CNT (0.0201 and 0.0195 s^−1^, respectively; Figure 1d and Table S2, Supporting Information), reflecting the formation of relatively more efficacious active sites for UOR. NiHyd@CNT demonstrates an ultralow Tafel slope of 21.6 mV dec^−1^ (Figure 1e), which is lower than that of α and β‐Ni(OH)_2_@CNT (40.8 and 56.2 mV dec^−1^, respectively) and several other reported catalysts, thereby highlighting the electrocatalytic superiority of NiHyd@CNT toward UOR (Table S3, Supporting Information). Additionally, NiHyd@CNT exhibits the highest double‐layer capacitance (*C_dl_*, Figure 1f and Figure S6a–c, Supporting Information), implying a larger electrochemically accessible surface area and, therefore, enhanced exposure of active sites, which subsequently results in enhanced electrocatalytic activity.

EIS is used to determine the faradaic charge‐transfer resistances – R_1_ and R_2_, which represent the resistances associated with the indirect and direct pathways of UOR, respectively (Figure 1g, Figure S6d–f, and Table S4, Supporting Information). The values of R_1_ and R_2_ (at 0.46 V versus Hg/HgO, for instance) are much smaller for NiHyd@CNT (14.0 and 37.0 Ω, respectively), followed by β‐Ni(OH)_2_@CNT (16.2 and 216.2 Ω, respectively), and α‐Ni(OH)_2_@CNT (59.5 and 235.9 Ω, respectively). The dramatically low charge transfer resistance of NiHyd@CNT further emphasizes its superior electrocatalytic prowess. For a clearer comparison of the overall catalytic performance, a spider plot is constructed, where a larger covered area indicates superior electrocatalytic properties (Figure 1h). The Nyquist plots of NiHyd (without CNT) at 0.50 V versus Hg/HgO in 0.33 m urea + 1 m KOH reveal that the charge transfer resistances (R_1_ and R_2_) for electrochemically activated NiHyd are 20.1 and 185.8 Ω, respectively, whereas these drop dramatically to 10.0 and 19.4 Ω for NiHyd@CNT (Figure S7a,b, Table S5, Supporting Information). This confirms that the CNT network facilitates faster interfacial electron transfer, which is crucial for the electrocatalytic process. Furthermore, the in situ growth of NiHyd on CNT ensures intimate interfacial contact, which reduces interfacial resistance and facilitates pre‐catalyst activation and conversion to the active γ‐NiOOH phase. This is also evident from the CVs shown in Figure S2e (Supporting Information), where pristine NiHyd exhibits limited UOR activity, while NiHyd@CNT hybrid demonstrates a drastic increase in current density, indicating a strong synergistic effect between the active catalyst and the conductive support.

Furthermore, step voltage analysis and RRDE measurements^[^ ^39^, ^40^, ^41^ ^]^ confirm that NiHyd@CNT exhibits high UOR current efficiency (85.6–99.2%, Figure 5i) and excellent selectivity toward UOR over OER in the potential range 0.45–0.70 V versus Hg/HgO (Figure S4, Supporting Information). The linear sweep voltammograms (LSV) recorded in 0.33 m U + 1 m KOH at 5 mV s^−1^ in a two‐electrode configuration show that overall urea‐assisted water electrolysis requires only 1.42 and 1.51 V to achieve current densities of 50 and 100 mA cm^−2^, respectively (Figure 1j). These potentials are 240 and 230 mV lower than the potentials required to achieve the same current densities (50 and 100 mA cm^−2^) from conventional water electrolysis (1.66 and 1.74 V in 1 m KOH, Figure 1j). Furthermore, the two‐electrode cell exhibits efficient hydrogen production when powered by a photovoltaic cell (see Videos S1 and S2, Supporting Information), highlighting its compatibility with renewable energy sources for sustainable green hydrogen generation.

The chronoamperometric stability test (*i‐t*) shows that NiHyd@CNT exhibits appreciable stability toward UOR at much higher current densities than the catalysts used for comparison (Figure 1k). It is clear from the *i‐t* curve that by the end of 42 h, the current density drops to 60% of the initial value, which could be due to deactivation of a few catalytic sites or due to periodic decrement in local urea concentrations at high current densities.^[^ ^42^ ^]^ To evaluate the long‐term stability of the CNT support under operational conditions, EIS measurements are conducted after prolonged chronoamperometry (Figure S7a,b, Table S5, Supporting Information). The charge transfer resistances are seen to increase only slightly (R_1_ = 13.8 Ω, R_2_ = 29.4 Ω), likely due to partial oxidation of the CNT at anodic potentials.^[^ ^43^ ^]^ Nevertheless, the modest change in resistance reveals the robust electrochemical and structural integrity of the NiHyd@CNT system during extended UOR electrolysis.

### 2.3 Understanding the Nature of the In Situ‐Formed Active Species

**Figure 2:**
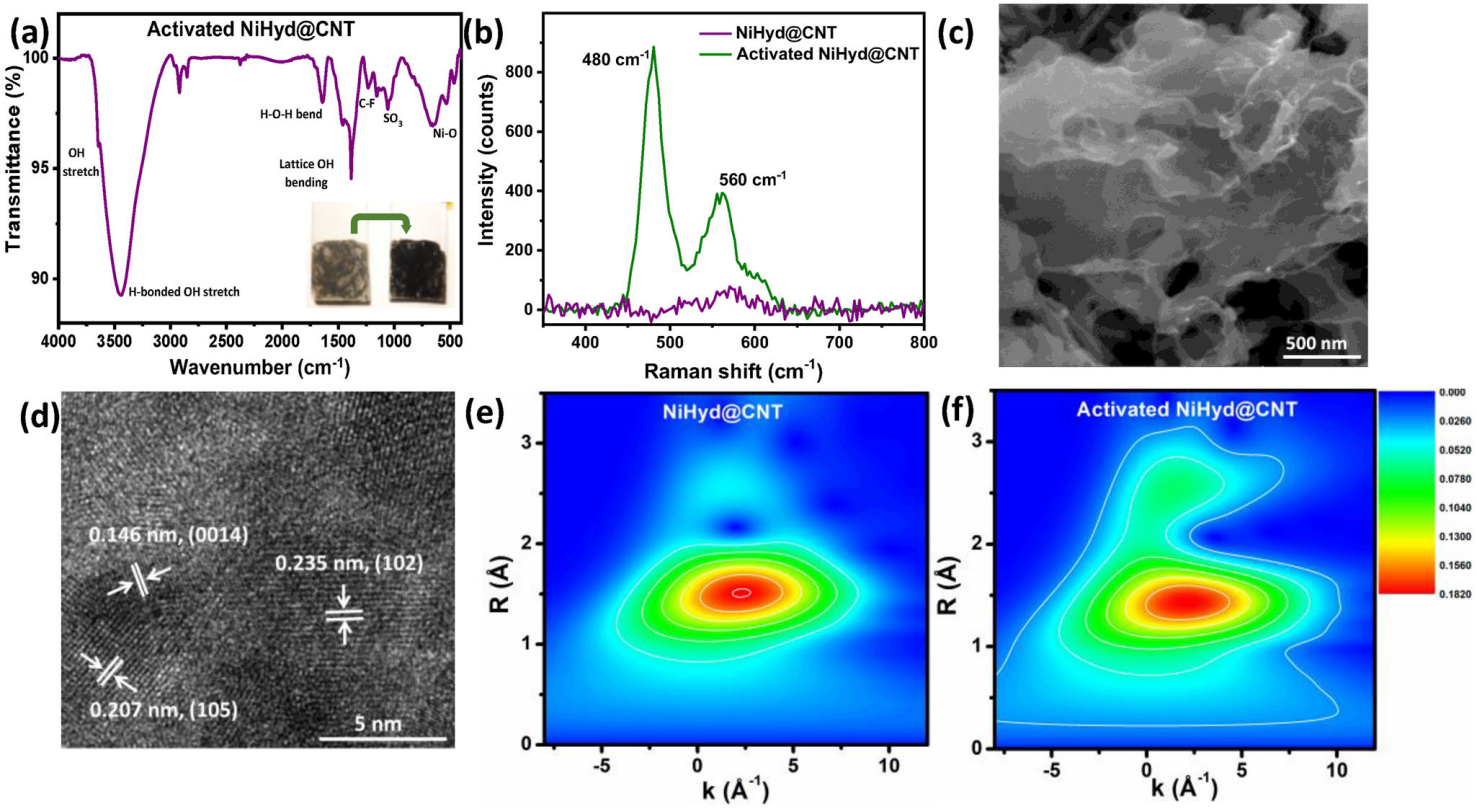
a) FTIR spectra of activated NiHyd@CNT (inset: optical image of FTO‐coated catalyst before and after electrochemical activation), b) Raman spectra of NiHyd@CNT, before and after activation, c,d) FESEM and HRTEM micrographs of activated NiHyd@CNT, respectively, Wavelet‐transform EXAFS of e) NiHyd@CNT, and f) Activated NiHyd@CNT.

The pre‐catalyst exhibits notable physicochemical changes after electrochemical activation. The color of the pre‐catalyst‐coated working electrode (F‐doped tin oxide (FTO)‐coated glass substrate) has changed from purple‐grey to black after electrochemical activation (inset of **Figure** **2**a). The powder XRD pattern of NiHyd@CNT has demonstrated a broad diffuse peak, indicating that the in situ formed catalyst could be X‐ray amorphous (Figure S8a, Supporting Information). The FTIR spectrum of NiHyd@CNT is shown in Figure 2a, and the stretching frequencies are assigned and tabulated in Table S1 (Supporting Information). The observed vibrations correspond to O–H stretching (both fundamental and H‐bonded), H–O–H bending, lattice O–H bending, in‐plane O–H deformation, and Ni–OH stretching confirm the complete transformation of the Ni complex (Refer to Figure S1b and Table S1, Supporting Information for comparison) into NiO_x_H_y_ species after the electrochemical activation step. The Raman spectra of NiHyd@CNT display two major Raman peaks at 470 and 556 cm^−1^, corresponding to the E_g_ bending vibration and the A_1g_ stretching vibration modes, respectively, of Ni–O in NiOOH (Figure 2b), thereby confirming the formation of NiOOH species.^[^ ^44^, ^45^ ^]^

FESEM micrographs of NiHyd@CNT illustrate the pronounced transformation of the pre‐catalyst into nanosheets of the “active phase” enmeshed in a CNT matrix (Figure 2c). Despite the X‐ray amorphous nature (Figure S8a, Supporting Information), high‐resolution TEM images (HRTEM) (Figure 2d) of activated NiHyd@CNT reveal lattice fringes with *d*‐spacings of 0.146, 0.207, and 0.235 nm, corresponding to the (0014), (105), and (102) planes of γ‐NiOOH (PDF No.: 06‐0075), respectively, suggesting localized crystallization of γ‐NiOOH in a few regions. The positive shift in the edge position of the Ni‐K edge X‐ray absorption near edge structure (XANES, Figure S8b, Supporting Information) suggests an appreciable increase in the oxidation state of Ni in the NiHyd@CNT^[^ ^46^ ^]^ due to the formation of Ni^3+^OOH species. The extended X‐ray absorption fine structure (EXAFS) of NiHyd@CNT‐PC (in R‐space) shows a peak at ≈2.052‐2.253 Å assigned to Ni‐N bonds in nickel hydrazine chloride (Figure S8c,d and Table S6, Supporting Information). After the reconstruction that accompanies electrochemical activation, Ni‐O and Ni‐Ni coordination shells emerge at 2.035 and 3.091 Å, respectively, which are characteristic of NiO_x_H_y_ phases (Figure S8d, Supporting Information).^[^ ^47^ ^]^ This transformation is made more apparent by comparing the wavelet‐transform (WT) of EXAFS (Figure 2e,f) that clearly shows the evolution of the WT signal for Ni–Ni bonds after electrochemical activation, thereby evidencing the aggregation of Ni species,^[^ ^48^ ^]^ which further corroborates our understanding that the pre‐catalyst transforms into NiOOH after electrochemical activation.

The hydrazine ligand in the Ni‐hydrazine complex plays a critical role specifically during the electrochemical activation step, rather than in the subsequent UOR itself. Hydrazine complexes of nickel are known to be unstable in alkaline media, undergoing rapid decomposition to form hydroxides of Ni due to the poor stability of the hydrazine ligand framework.^[^ ^49^ ^]^ This inherent lability is strategically exploited under anodic polarization to accelerate the in situ generation of NiOOH species. Importantly, once the transformation is complete, the bulk of the pre‐catalyst no longer retains the hydrazine ligands, indicating that these ligands have no direct role in UOR catalysis. Instead, their primary contribution lies in modulating the kinetics and structural dynamics of the activation process. To further assess the advantage of hydrazine ligands in facilitating NiOOH formation, we have compared the UOR activity of NiHyd@CNT with other nickel compounds coordinated by different ligands; nickel phthalocyanine, where Ni is chelated by four nitrogen donors,^[^ ^50^ ^]^ and nickel oxalate, coordinated by two bidentate oxalate ligands and two H_2_O molecules.^[^ ^51^ ^]^ Both Ni phthalocyanine@CNT and Ni oxalate@CNT have shown significantly lower UOR activity after electrochemical activation, highlighting that the unique lability of hydrazine‐coordination accelerates the restructuring and formation of the active NiOOH phase more effectively (Figure S5, Supporting Information).

### 2.4 Studying Regeneration of Active Species: In situ Raman Spectroscopy

To gain deeper insights into the dynamic changes occurring during the electrochemical process, in situ Raman spectra are acquired under electrochemical conditions (experimental setup in Figure S9a, Supporting Information). A UOR‐induced NiOOH‐depletion Raman experiment was designed to track the dynamic evolution of NiOOH species. In this experiment, a fixed anodic potential is applied briefly to generate NiOOH, followed by holding the electrode at open‐circuit potential (OCP) for 180 s before acquiring Raman spectra. The potential is then reapplied to monitor the reversible disappearance and reappearance of NiOOH peaks, revealing the consumption and regeneration dynamics of these species in the presence of urea. According to the indirect UOR mechanism, the electrochemically formed NiOOH species chemically oxidize urea into the oxidation products, and in this process, the active NiOOH species get converted (are consumed) to Ni(OH)_2_.^[^ ^52^, ^53^ ^]^ Subsequently, the generated Ni(OH)_2_ species need to be re‐oxidized electrochemically into NiOOH to sustain the next cycle of UOR. Thus, the attenuation or loss of NiOOH Raman peaks within the detection capabilities of the Raman measurement indicates their chemical consumption during UOR, while their reappearance upon reapplication of potential reflects their electrochemical regeneration.

**Figure 3:**
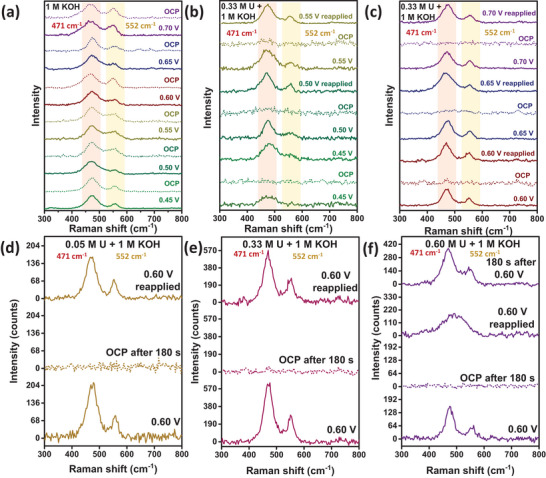
In situ Raman spectra of NiHyd@CNT acquired in (a) 1 m KOH, b,c,e) 0.33 m urea + 1 m KOH, d) 0.05 m urea + 1 m KOH, f) 0.60 m urea + 1 m KOH. All spectra at open‐circuit potential (OCP) are recorded after holding at OCP for 180 s.

The influence of applied potential and urea concentration on the consumption‐regeneration dynamics of UOR active species‐NiOOH in the case of NiHyd@CNT is studied in detail. Initially, control experiments are performed in 1 m KOH without urea to check the stability of NiOOH. As shown in **Figure** **3**a, two characteristic peaks at 471 and 552 cm^−1^ corresponding to the E_g_ mode and A_1g_ modes, respectively, of the NiO_2_ framework in NiOOH species,^[^ ^54^ ^]^ remain intact at all potentials (0.45 to 0.70 V) after 180 s under OCP in 1 m KOH electrolyte. However, for the urea‐containing KOH electrolyte, at 0.45 V, the NiOOH peaks are significantly unresolved in the presence of urea, indicating poor regeneration of NiOOH even under applied potential (Figure 3b,c). After a 180 s OCP hold, reapplication of 0.45 V yielded only minimal peak recovery, suggesting sluggish regeneration at this potential. In contrast, at higher applied potentials (0.50–0.70 V), NiOOH peaks that diminish during OCP rapidly re‐emerge once the potential is reapplied. This demonstrates the high regenerability of NiOOH formed from the designed pre‐catalyst when subjected to sufficiently elevated anodic potentials.

The influence of urea concentration on the regeneration of active species is further investigated at 0.60 V in 0.05, 0.33, and 0.60 m urea electrolytes (in 1 m KOH, Figure 3d–f). While 0.05 and 0.33 m urea conditions show rapid peak recovery after OCP, 0.60 m urea required ≈6 min for full regeneration. This delay indicates that at higher urea concentrations, the NiOOH regeneration is impeded, likely due to an increased reaction demand.

Overall, these results suggest that while applied potential accelerates NiOOH regeneration, urea concentration exerts a stronger influence on the steady‐state NiOOH population within the tested range.

### 2.5 Oxidation State Dynamics of Ni During UOR: In situ XAS 

**Figure 4:**
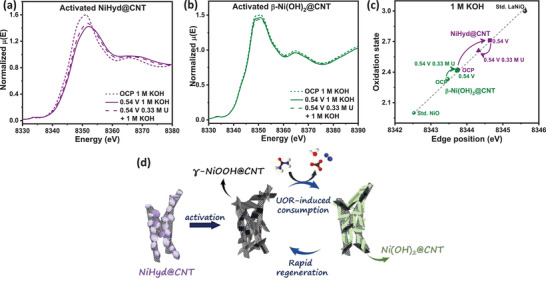
In situ Ni K‐edge XANES plot of a) NiHyd@CNT, b) β‐Ni(OH)_2_@CNT, c) Calibration curve for calculation of the oxidation state of Ni from the Ni K‐edge positions, d) Schematic illustration of the electrochemical activation process and dynamic changes in active species during UOR.

In order to quantitatively monitor the dynamic changes in the oxidation state of Ni in the catalyst during UOR, XANES spectra are acquired under applied potential in a suitable electrolyte using the in situ XAS cell setup shown in Figure S9b (Supporting Information). The average oxidation state of Ni under electrochemical operating conditions is calculated using the edge positions of in situ acquired Ni K‐edge XANES spectra of NiHyd@CNT and β‐Ni(OH)_2_@CNT (**Figure** **4**a,b, Figure S10a–c, and Table S7, Supporting Information). The in situ XANES spectra of NiHyd@CNT and β‐Ni(OH)_2_@CNT, and the ex‐situ XANES spectra of reference standards – NiO (Ni^2+^) and LaNiO_3_ (Ni^3+^) are shown in Figure S10a (Supporting Information). Figure 4a–c and Figure S10a–c (Supporting Information) show that when an anodic potential is applied (in 1 m KOH electrolyte), the edge position of Ni K‐edge shifts positively with respect to that of the XANES spectra acquired under OCP. This shift in edge position is due to an increase in the oxidation state of Ni, under applied anodic potential, due to the formation of high valent Ni‐containing species‐NiOOH.^[^ ^55^ ^]^ The edge position of NiHyd@CNT at OCP shifts significantly to higher energy at 0.54 V (8343.8 eV → 8344.6 eV). Using the integral method of calculation of edge positions and from the calibration curve obtained from the formal oxidation states of Ni in the reference compounds (Figure 4c and Table S7, Supporting Information),^[^ ^56^, ^57^ ^]^ it is calculated that the average oxidation state of Ni increases from 2.42 (activated NiHyd@CNT at OCP) to 2.71 (activated NiHyd@CNT at 0.54 V). However, in the case of activated β‐Ni(OH)_2_@CNT, the edge position shift is subtle, i.e., 8343.5 eV (at OCP) to 8343.7 eV (at 0.54 V), which corresponds to an average Ni oxidation state change from 2.33 to 2.42. The shift in edge position on the application of anodic potential for NiHyd@CNT (0.8 eV) is more pronounced than the shift in edge position of activated β‐Ni(OH)_2_@CNT (0.2 eV). This is indicative of the increased oxidation degree of Ni in NiHyd@CNT when compared to activated β‐Ni(OH)_2_@CNT.^[^ ^58^ ^]^ In other words, NiHyd@CNT furnishes an abundance of NiOOH active species on anodic polarization when compared to activated β‐Ni(OH)_2_@CNT. Furthermore, the XANES spectra are also acquired in the presence of urea (0.33 m U + 1 m KOH), to monitor changes in the oxidation state during urea electrolysis. From Figure 4c and Figure S10b (Supporting Information), it is evident that the edge position of NiHyd@CNT demonstrates a negative shift from 8344.6 eV (at 0.54 V in 1 m KOH) to 8344.3 eV (at 0.54 V in 0.33 m U + 1 m KOH) on the addition of urea. This indicates that the average oxidation state of Ni decreases in the presence of urea (2.71 → 2.61). This phenomenon, as also documented in several reported Ni‐based UOR electrocatalysts, occurs due to the rapid consumption of NiOOH species as a consequence of the indirect mechanism of UOR.^[^ ^39^, ^48^ ^]^ The degree of consumption of in situ generated NiOOH species is shown to reflect the effectiveness of the catalyst in oxidizing urea.^[^ ^39^ ^]^ The edge position shift for NiHyd@CNT in the presence of urea is 0.3 eV. In comparison, there is no perceivable shift in the edge position of activated β‐Ni(OH)_2_@CNT on the introduction of urea (E_edge_ = 8343.7 eV, Figure 4c and Figure S10c, Supporting Information). This indicates that UOR‐induced consumption of NiOOH sites is more prominent in the case of NiHyd@CNT, signifying better UOR output. This observation aligns with the high electrocatalytic activity parameters demonstrated by the activated NiHyd@CNT. Furthermore, although there is a considerable negative shift in the edge position for activated NiHyd@CNT, the average oxidation state of Ni in the presence of urea (2.61) does not drop below that of the activated catalyst at OCP (2.42) (Figure 4c). As also gleaned from in situ Raman studies, this indicates that perhaps there is continuous and rapid replenishment of NiOOH active species in NiHyd@CNT, which is essential to sustain UOR electrolysis over long periods, even at high current densities.

In situ Raman and XAS analyses reveal that NiHyd@CNT undergoes a higher degree of electrochemical oxidation to form abundant NiOOH species under anodic polarization, which are rapidly consumed and efficiently regenerated during urea electrolysis, which is critical for sustained UOR activity (Figure 4d).

### 2.6 AEM Urea‐Assisted Hybrid Water Electrolyzer Studies

**Figure 5:**
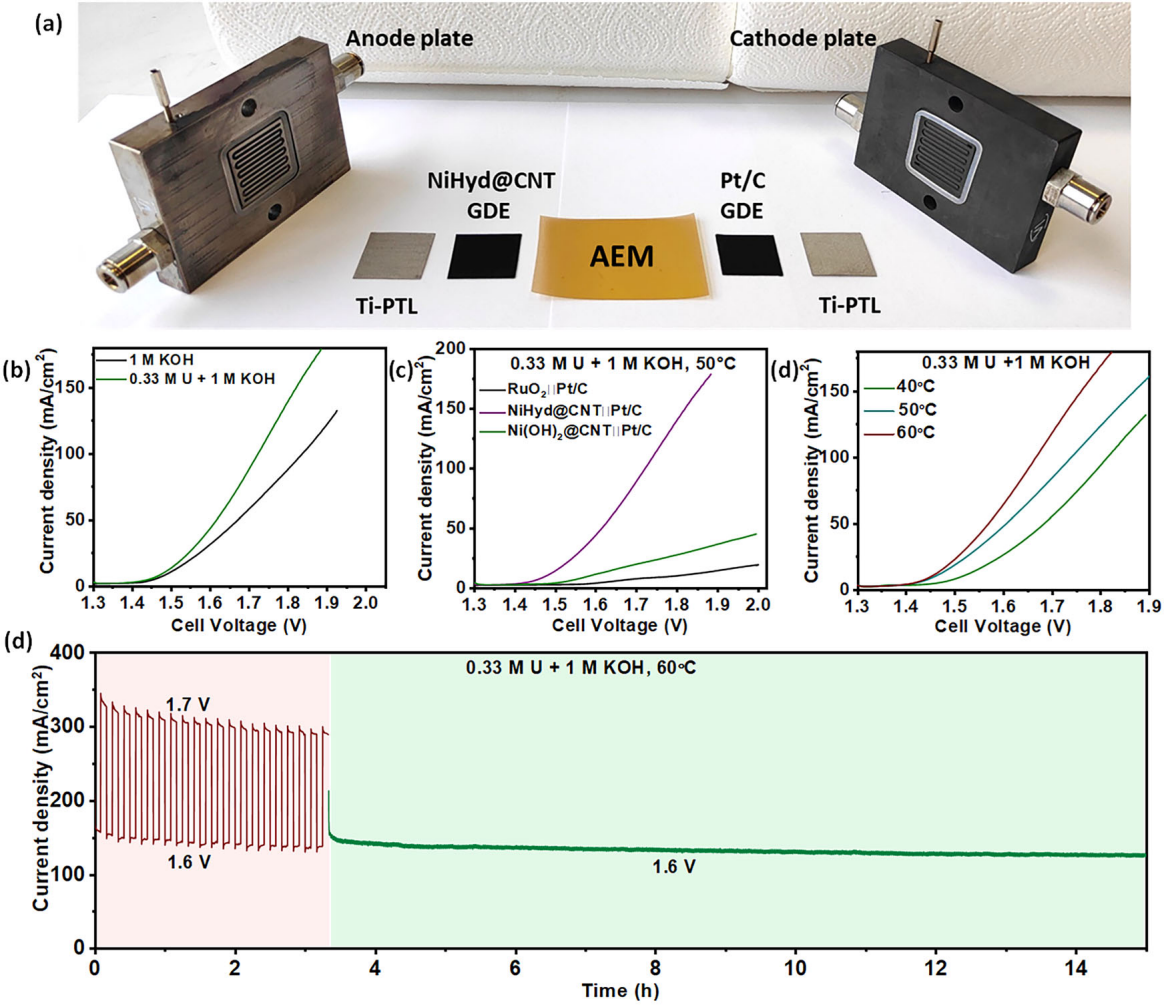
a) Photographs of AEM electrolyzer assembly, IV curves of b) NiHyd@CNT (on carbon fiber paper (CFP))||Pt/C in 1 m KOH and 0.33 m urea + 1 m KOH, c) RuO_2_ (on CFP)/NiHyd@CNT /Ni(OH)_2_@CNT||Pt/C, d) Electrolyzer stability test of NiHyd@CNT (on Ni felt)||Pt/C (all IVs are 100% iR‐corrected).

To mimic real‐world conditions, catalyst performance was evaluated using a zero‐gap single‐cell AEM electrolyzer (**Figure** **5**a). The *I–V* polarization curve of NiHyd@CNT||Pt/C electrolyzer for urea‐assisted water electrolysis demonstrates that a current density of 100 mA cm^−2^ is achieved at a cell voltage that is 120 mV lower than that of conventional alkaline water electrolysis (OER coupled with HER, (Figure 5b and Figure S11a, Supporting Information).

Furthermore, the *I–V* curves shown in Figure 5c demonstrate that the NiHyd@CNT||Pt/C urea electrolyzer exhibits much higher current density at lower overpotentials when compared to the conventional electrolyzer assemblies‐Ni(OH)_2_@CNT||Pt/C and RuO_2_||Pt/C, thereby highlighting the superiority of the NiHyd@CNT system for urea‐assisted water splitting (Figure S11b,c,d, Supporting Information). Increasing the operating temperature from 40 to 60 °C reduces the cell potential at 100 mA cm^−2^ by ≈140 mV (Figure 5d and Figure S11c, Supporting Information), which could be due to improved reaction kinetics, electrolyte conductivity, and gas removal at higher temperatures.^[^ ^59^ ^]^

The AEM electrolyzer performance under cell voltage switching shows ≈87% and ≈90% retention of the initial current density at 1.6 and 1.7 V, respectively, when subjected to 20 switching cycles (Figure 5e). Impressively, in the following amperometric stability test at 1.6 V, the electrolyzer retained 82% of its initial (stabilized) current density after 12 h of electrolysis, demonstrating robust performance. The *I–V* curve obtained after the stability test shows only a modest decrease in current density compared to that recorded before the test, indicating stable performance (Figure S11e, Supporting Information). The Nyquist plots obtained from potentiostatic EIS measurements at 1.6 V, before and after the stability tests, show no significant changes in the uncompensated and charge transfer resistances, confirming the stability of the AEM electrolyzer (Figure S11f, Supporting Information).^[^ ^60^ ^]^ The fabricated AEM electrolyzer is calculated to demonstrate an efficiency of 70.4% at a current density of 0.5 A/cm^2^ (refer to “AEM electrolyzer studies” section in Supporting Information).^[^ ^61^, ^62^ ^]^

While this study establishes the concept and mechanistic understanding of Ni‐based pre‐cah;talyst activation pathways and demonstrates their integration potential in AEM electrolyzers, the output current density could be further boosted by employing binder‐free architectures to reduce resistance and mass‐transport losses.^[^ ^63^, ^64^ ^]^

### 2.7 Post‐Stability Characterization

Post‐stability FESEM micrographs of the catalyst show that the catalyst retains the morphology of the activated phase (Figure S12a, Supporting Information). HRTEM micrographs (Figure S12b,c, Supporting Information) of the post‐stability samples of the catalyst demonstrate lattice fringes that correspond to (0014), (105), and (102) planes of γ‐NiOOH, and (200) and (300) planes of 3Ni(OH)_2_.2H_2_O (PDF no.: 22‐0444). X‐ray photoelectron spectroscopy (XPS) is  performed to ascertain the chemical nature of the surface of the catalyst after the prolonged stability test. The deconvoluted high‐resolution Ni 2p spectrum of NiHyd@CNT after the stability test (15 h, Figure S12d, Supporting Information) shows peaks at 855.5 and 873.1 eV, corresponding to Ni2p_3/2_ and Ni2p_1/2_ of Ni^2+^, and peaks at 856.6 and 874.4 eV corresponding to Ni2p_3/2_ and Ni2p_1/2_ of Ni^3+^. The characteristic satellite peaks are also observed at 861.9, 866.6, and 880.0 eV, respectively.^[^ ^65^ ^]^ The Ni^3+^/Ni^2+^ areal ratio for NiO was calculated to be 3.10. Deconvoluted high‐resolution O 1s spectrum of NiHyd@CNT after the stability test shows peaks centred ≈531.2, ≈533.1, and ≈534.9 eV labelled as – O_1_, O_2_, and O_3_, respectively (Figure S12e, Supporting Information). The peak O_1_ corresponds to lattice oxygen in metal oxides, O_2_ originates from surface oxygen atoms passivated with adsorbed hydrogen (hydroxyl species) or surface chemisorbed oxygen such as O_2_^2–^ or O^–^, and O_3_ is attributed to adsorbed H_2_O.^[^ ^66^, ^67^, ^68^ ^]^ The appearance of lattice fringes corresponding to 3Ni(OH)_2_·2H_2_O along with NiOOH in HRTEM images and the coexistence of Ni^2+^ and Ni^3+^ ions observed in XPS for the post‐stability NiHyd@CNT sample, can be attributed either to the partial conversion of NiOOH into Ni(OH)_2_ via the indirect UOR mechanism or to phase changes induced by exposure to ambient atmosphere during ex situ analysis.^[^ ^46^ ^]^ Nevertheless, the in situ Raman and XAS experiments confirm the efficient regeneration of γ‐NiOOH during operation, suggesting that this dynamic redox cycling—although inherent to the indirect mechanism—does not critically compromise long‐term stability as long as regeneration remains efficient. However, any loss or degradation of active sites during regeneration, by Ni leaching or local deactivation of the in situ (re‐)generated species, could influence the long‐term stability of the catalyst. To assess the chemical stability of the catalyst, inductively coupled plasma mass spectrometry (ICP‐MS) analysis of the spent electrolyte after 48 h of electrolysis is performed, which revealed only 11.56 ppb of Ni, corresponding to 0.031% Ni leaching (based on 15 mL electrolyte and 0.56 mg catalyst loading). This confirms negligible dissolution of active Ni sites, indicating that the catalyst retains its Ni content.

Fitting of EXAFS spectra in R space highlighted significant changes in both the Ni‐O and Ni‐Ni coordination shells after the prolonged stability test (Figure S8e,f and Table S6, Supporting Information). The Ni‐O bond length increased from 2.036 Å (NiHyd@CNT) to 2.053 Å (post‐stability), suggesting a decrease in the average oxidation state of Ni,^[^ ^46^ ^]^ likely due to the coexistence of NiOOH and Ni(OH)_2_ phases post‐stability. Additionally, the Ni‐Ni coordination number increased from 4.08 (±0.45, NiHyd@CNT) to 5.74 (±0.52, post‐stability) after the stability test, indicating catalyst reconstruction and subsequent curing of Ni vacancies, which could have an adverse effect on electrocatalytic activity, as it reduces the density of active sites available for the reaction.^[^ ^36^, ^69^ ^]^ Therefore, in addition to local urea depletion, the gradual current decay observed in Figure 1k may be attributed to deactivation of active species via vacancy healing.

## 3 Conclusion

A hydrazine complex of Ni enmeshed in a conducting CNT matrix is demonstrated as a pre‐catalyst for UOR, which, upon electrochemical activation, rapidly reconstructs into highly active γ‐NiOOH species that are continuously consumed and effectively regenerated during urea electrolysis. In situ Raman and XANES analyses reveal that the activated catalyst sustains rapid, potential‐dependent formation of NiOOH species during UOR, maintaining a sufficiently high steady‐state NiOOH population. This efficient regeneration not only drives exceptional activity and durability but also highlights the importance of dynamic active‐species renewal as a principle for UOR catalyst design. The pre‐catalyst, when employed as the anode in an AEM electrolyzer, successfully demonstrated efficient urea‐assisted water electrolysis under operationally relevant conditions. This study answers the central questions posed in the introduction of the manuscript and highlights the potential of Ni‐based coordination complexes as pre‐catalysts for anodic oxidation reactions that fully transform into active electrocatalytic phases. These findings emphasize the significance of simple, self‐reconstructing pre‐catalysts as an efficient strategy for developing high‐performance electrocatalysts, possibly for large‐scale applications.

## 4 Experimental Section

### Synthesis of Ni Hydrazine Complex@C‐Additive

NiHyd@CNT‐PC was synthesized using a simple chemical route. 0.50 g of Nickel (II) chloride hexahydrate (Sigma Aldrich) was dissolved in ethanol solution by bath sonication, and ‐COOH‐functionalized multiwalled carbon nanotubes (MWCNTs, Jiangsu Cnano Technology Co., Ltd., avg. diameter 10‐25 nm) were added to this solution. The solution was sonicated till the MWCNTs were completely dispersed. The reaction vessel was then placed in an oil bath maintained at a temperature of 80 °C, and 1 mL of hydrazine hydrate (FINAR, 99‐100%) was added drop‐wise to this solution under continuous stirring at a temperature of 80 °C for 45 min. A purple‐grey‐coloured precipitate was formed, which was then washed several times with ethanol by centrifugation. The compound was then dried at room temperature (25 °C), finely pounded into a fine powder using an agate mortar and pestle,‐ and stored in plastic vials in a vacuum desiccator. A similar procedure was employed for the synthesis of NiHyd@rGO‐PC and NiHyd@VC‐PC by replacing MWCNT with reduced graphene oxide (rGO) and Vulcan carbon XC72, respectively.

### Characterization

XRD patterns were obtained from a Rigaku SmartLab X‐ray diffractometer using Cu Kα radiation (λ = 1.5406 Å) in the Bragg‐Brentano focusing mode with θ increment of 1.2 °/min. High‐resolution surface images of the catalysts were obtained from TESCAN MIRA3 LMU FESEM. TEM images and selected area electron diffraction (SAED) patterns were captured using a Thermo Scientific Talos F200S G2 TEM instrument. Fourier Transform Infrared (FTIR) Spectroscopy was performed using Perkin Elmer Spectrum 3 with a deuterated triglycine sulfate (DTGS) detector. To accurately determine the amount of Ni in the coordination complex, ICP‐OES was performed using the Thermo Scientific Duo iCAP 6000 series, and ICP‐MS was performed using the NexION 2000 (PerkinElmer, USA). To determine the amount of N and H in the complex, an Elemental Analyser – Perkin Elmer CHNS/O 2400 was used. The amount of Cl in the complex was determined using silver nitrate‐precipitation titrimetry. XPS measurements were performed using an EnviroESCA instrument (SPECS) equipped with a monochromated Al Kα X‐ray source (photon energy = 1486.71 eV) and a Phoibos 160 NAP 1D‐DLD hemispherical analyzer. The analyzed spot size was ≈300 µm in diameter. To mitigate sample charging, argon gas was introduced at a flow rate of 25 mL min^−1^, and all measurements were conducted at a chamber pressure of 2 mbar. The high‐resolution XPS spectra were deconvoluted and fitted using the XPSPEAK41 software, with a linear background function, and charging correction was done by levelling the C1s spectrum to 284.8 eV.

XAS experiments at room temperature (298 K) were performed at PETRA III, P64 beamline of Deutsches Elektronen‐Synchrotron (DESY), Germany.^[^ ^70^ ^]^ Ex‐situ XAS samples were prepared by mixing the material with boron nitride powder and then pressing them into pellets, which were then used to acquire XAS spectra in the transmission mode. In situ XAS measurement was performed using a custom‐made acrylate‐based, spectro‐electrochemical cell (Figure S9b, Supporting Information) fitted with a Kapton window that was transparent to X‐rays and catalyst‐coated CFP positioned close to the window (ca. 0.2 mm distance) to minimize the volume of electrolyte between the Kapton window and the catalyst surface. Ni‐K edge spectra were acquired in fluorescence mode with a Si (111) double crystal monochromator and passivated implanted planar diode (PIPS) detector. ATHENA software was used to perform background subtraction, normalization, and alignment of XANES. The integral method^[^ ^56^ ^]^ was used for calculating the edge positions of Ni‐K edge XANES spectra, and the average oxidation states of Ni were calculated using the calibration curve constructed from the ex‐situ XANES spectra of reference standards – NiO (Ni^2+^) and LaNiO_3_ (Ni^3+^). ARTEMIS software was used for fitting the EXAFS data. In situ Raman spectra were acquired using Horiba Jobin Yvon XploRA PLUS V1.2 MULTILINE (532 nm laser with 50 X, laser power of 6.25 mW, accumulation of 2 cycles, and acquisition time of 60 s. A custom‐made acrylate‐based spectro‐electrochemical cell with a window for entry of the Raman laser was used during the in situ Raman studies (Figure S9a, Supporting Information). In situ XANES and Raman measurements were acquired from the catalysts that were coated on CFP after obtaining a steady current at the desired potential after electrochemical activation.

### Electrochemical Studies–Three‐Electrode Measurements

All electrochemical studies were carried out using a CH760 electrochemical workstation on a glassy carbon electrode (GCE), with Pt wire as the counter and Hg/HgO as the reference electrode. 2 mg of the catalyst was dispersed in a solution containing 150 µL ultrapure water, 15 µL of *N,N‐*dimethylformamide (≥99.9%, Sigma‐Aldrich), 35 µL of isopropanol, and 50 µL (2 wt.%) Nafion (Sigma‐Aldrich) solution by sonication for 30 min to obtain a homogeneous catalyst ink. 5 µL of the ink was drop‐cast onto a well‐polished, clean GCE of surface area 0.07 cm^2^ and used as the working electrode (catalyst mass loading of 40 µg). Cyclic voltammetry (CV) was performed at various scan rates in the potential window of 0 V to 0.7 V versus Hg/HgO in urea – KOH solutions as the electrolyte. To nullify the influence of the conducting additive, the α and β‐Ni(OH)_2_ catalysts were also supplemented with CNT during electrode preparation and subjected to the same activation protocol (Figure S2b,c, Supporting Information). Electrochemical impedance spectroscopy (EIS) was performed in a frequency range of 10^4^ to 10^−1^ Hz with the amplitude of the sinusoidal wave set to ±5 mV. The chronoamperometric stability test (*i‐t*) was performed at 0.60 V versus Hg/HgO in a three‐electrode configuration in 0.33 m urea + 1 m KOH electrolyte for all three catalysts on an L‐shaped GCE to dislodge the bubbles of gaseous products formed during UOR.

### Electrochemical Studies–Two‐Electrode Measurements

The urea electrolysis was also performed in a two‐electrode configuration to assess the reduction in cell voltage for H_2_ generation, realized by replacing OER with UOR. Two‐electrode measurements were performed with an L‐shaped glassy carbon electrode modified with NiHyd@CNT as the anode and a platinum (Pt) coil as the cathode (NiHyd@CNT||Pt configuration) and compared with that of a similarly assembled RuO_2_ ||Pt configuration in 0.33 m urea in 1 m KOH electrolyte.

### Electrochemical Studies–AEM Electrolyzer Assembly and Testing

Biologic SP150 potentiostat with 10 A booster was used for electrolyzer experiments. LeanCat air‐pressed single cell with an active area of 2.2 x 2.2 cm^2^, equipped with PID‐controlled heating on the anode and cathode, was used to test the performance of the developed pre‐catalyst in an AEM electrolyzer. The anode comprises the pre‐catalyst (NiHyd@CNT‐PC) coated on CFP, and nickel felt, and a Pt/C gas diffusion electrode (GDE) with a Pt loading of 0.5 mg cm^−2^ was used as the cathode. Platinized Ti mesh was used as the porous transport layer (PTL) on both the anode and cathode. The AEMs – Fumacep‐FAA‐PK‐130 and PiperION were used after conditioning the membranes in 1 m and 0.50 m KOH solutions, respectively. 1 m KOH and 0.33 m U + 1 m KOH electrolyte were flowed on the cathode and anode sides, respectively, at a flow rate of 5 mL min^−1^. The dependence of electrolyzer performance on the temperature of the electrolyzer was tested between 40 and 60 °C.

Stability of the catalyst was monitored using the AEM electrolyzer with NiHyd@CNT coated on more resilient‐Ni fibre felt as the anode, as CFP may get degraded at anodic potential and high current passage.^[^ ^71^ ^]^ AEM electrolyzers were also assembled with NiHyd@CNT‐coated Ni felt||Pt/C, uncoated‐Ni felt||Pt/C, and Ni(OH)_2_@CNT‐coated Ni felt||Pt/C (for activity comparison), and the *I–V* curves were compared with that of NiHyd@CNT‐coated Ni felt||Pt/C (Figure S11d, Supporting Information). The operating temperature that was chosen for the stability test was 60 °C, and PiperION AEM (30 µm thickness) was used to withstand these conditions for extended time periods. The stability of the cell was monitored by varying the cell voltage at 1.6 and 1.7 V at periodic intervals (5 min each) to emulate the fluctuations expected when the electrolyzer was powered by photovoltaic sources. This was followed by an amperometric stability test recorded at a cell voltage of 1.6 V for 12 h.

## Conflict of Interest

The authors declare no conflict of interest.

## Author Contributions

N.N.R. conceived the idea, designed and conducted the experiments, analysed the data, and drafted the original manuscript. A.A.K. and P.K. designed and conducted the experiments. C.A., M.S.N.K., and T.H. contributed to X‐ray absorption and X‐ray photoelectron spectroscopy experiments. P.K. contributed to electrolyzer experiments. P.K. and I.M. acquired funds for the study and reviewed the manuscript. N.S.J. acquired funds for the study, supervised, validated the results, reviewed and edited the manuscript.

## Supporting information



Supporting Information

Supplemental Video 1

Supplemental Video 2

## Data Availability

The data that support the findings of this study are available from the corresponding author upon reasonable request.

## References

[1] B. K. Boggs , R. L. King , G. G. Botte , Chem. Commun. 2009, 4859. 10.1039/b905974a 19652805

[2] X. Hu , J. Zhu , J. Li , Q. Wu , ChemElectroChem 2020, 7, 3211.

[3] D. A. Daramola , D. Singh , G. G. Botte , The Journal of Physical Chemistry A. 2010, 114, 11513. 20936868 10.1021/jp105159t

[4] J. Gallenberger , H. Moreno Fernández , A. Alkemper , M. Li , C. Tian , B. Kaiser , J. P. Hofmann , Catal. Sci. Technol. 2023, 13, 4693.

[5] J. Feng , X. Wang , H. Pan , Adv. Mater. 2024, 36, 2411688. 39436113 10.1002/adma.202411688 PMC11635912

[6] W. Sun , J. Li , W. Gao , L. Kang , F. Lei , J. Xie , Chem. Commun. 2022, 58, 2430. 10.1039/d1cc06290e 35084411

[7] L. Xia , B. F. Gomes , W. Jiang , D. Escalera‐López , Y. Wang , Y. Hu , A. Y. Faid , K. Wang , T. Chen , K. Zhao , X. Zhang , Y. Zhou , R. Ram , B. Polesso , A. Guha , J. Su , C. M. S. Lobo , M. Haumann , R. Spatschek , S. Sunde , L. Gan , M. Huang , X. Zhou , C. Roth , W. Lehnert , S. Cherevko , L. Gan , F. P. García de Arquer , M. Shviro , Nat. Mater. 2025, 24, 753. 40021826 10.1038/s41563-025-02128-7

[8] X. Liu , R. Guo , W. Huang , J. Zhu , B. Wen , L. Mai , Small 2021, 17, 2100629. 10.1002/smll.202100629 34288417

[9] M. Ajmal , S. Zhang , X. Guo , X. Liu , C. Shi , R. Gao , Z.‐F. Huang , L. Pan , X. Zhang , J.‐J. Zou , Appl. Catal. B: Environ. and Energy 2025, 361, 124561.

[10] S. Kumar Pal , B. Singh , J. K. Yadav , C. L. Yadav , M. G. B. Drew , N. Singh , A. Indra , K. Kumar , Dalton Trans. 2022, 51, 13003. 35968800 10.1039/d2dt01971j

[11] S. K. Pal , T. Ansari , C. L. Yadav , N. Singh , P. Lama , A. Indra , K. Kumar , Dalton Trans. 2025, 54, 1597. 39660446 10.1039/d4dt02447h

[12] H. Zhang , P. Li , S. Chen , F. Xie , D. J. Riley , Adv. Funct. Mater. 2021, 31, 2106835.

[13] M. M. Najafpour , Inorg. Chem. 2025, 64, 3079. 39895212 10.1021/acs.inorgchem.4c05397

[14] Y. Shi , Y. Yu , Y. Liang , Y. Du , B. Zhang , Angew. Chem., Int. Ed. 2019, 58, 3769. 10.1002/anie.201811241 30549367

[15] P. W. Menezes , S. Yao , R. Beltrán‐Suito , J. N. Hausmann , P. V. Menezes , M. Driess , Angew. Chem., Int. Ed. 2021, 60, 4640. 10.1002/anie.202014331 PMC7986911 33169889

[16] W. Zheng , L. Y. S. Lee , ACS Energy Lett. 2021, 6, 2838.

[17] V. Maruthapandian , S. Kumaraguru , S. Mohan , V. Saraswathy , S. Muralidharan , ChemElectroChem 2018, 5, 2795.

[18] M. Yuan , R. Wang , Z. Sun , L. Lin , H. Yang , H. Li , C. Nan , G. Sun , S. Ma , Inorg. Chem. 2019, 58, 11449. 31397562 10.1021/acs.inorgchem.9b01124

[19] J. Y. Luo , Y. Yuan , H. Y. Ruan , X. Q. Wu , Y. P. Wu , S. Li , G. Zhang , S. Sun , D. S. Li , Small Struct. 2023, 4, 2300074.

[20] S. Zhao , C. Tan , C. T. He , P. An , F. Xie , S. Jiang , Y. Zhu , K. H. Wu , B. Zhang , H. Li , J. Zhang , Y. Chen , S. Liu , J. Dong , Z. Tang , Nat. Energy 2020, 5, 881.

[21] Y. Mousazade , M. R. Mohammadi , P. Chernev , R. Bagheri , Z. Song , H. Dau , M. M. Najafpour , Inorg. Chem. 2020, 59, 15335. 33021376 10.1021/acs.inorgchem.0c02305

[22] J. Kim , M. Kim , S. S. Han , K. Cho , Adv. Funct. Mater. 2024, 34, 2315625.

[23] G. Moon , M. Yu , C. K. Chan , H. Tüysüz , Angew. Chem., Int. Ed. 2019, 58, 3491. 10.1002/anie.201813052 30664307

[24] T. Kahlstorf , J. Niklas Hausmann , I. Mondal , K. Laun , I. Zebger , T. Sontheimer , P. W. Menezes , Green Chem. 2023, 25, 8679.

[25] J. W. Park , E. H. Chae , S. H. Kim , J. H. Lee , J. W. Kim , S. M. Yoon , J.‐Y. Choi , Mater. Chem. Phys. 2006, 97, 371.

[26] C. Gao , Z. Lu , Y. Yin , Langmuir 2011, 27, 12201. 21861481 10.1021/la203196a

[27] H. Qin , Y. Ye , J. Li , W. Jia , S. Zheng , X. Cao , G. Lin , L. Jiao , Adv. Funct. Mater. 2023, 33, 2209698.

[28] B. Song , Y. Feng , J. Su , L. Li , Q. Shao , J. Lu , ACS Appl. Nano Mater. 2023, 6, 23142.

[29] W. Yang , X. Yang , B. Li , J. Lin , H. Gao , C. Hou , X. Luo , J. Mater. Chem. A 2019, 7, 26364.

[30] X. Dong , C. Peng , X. Zhao , T. Zhang , Y. Liu , G. Xu , J. Zhou , F. Guo , Z. Yu , X. Jia , RSC Adv. 2023, 13, 29625. 37822661 10.1039/d3ra05538h PMC10562896

[31] Z. Cao , H. Mao , X. Guo , D. Sun , Z. Sun , B. Wang , Y. Zhang , X.‐M. Song , ACS Sustainable Chem. Eng. 2018, 6, 15570.

[32] Y. Li , F. Luo , Y. Xie , C. Chang , M. Xie , Z. Yang , Int. J. Hydrogen Energy 2023, 48, 9155.

[33] L. Xia , Y. Liao , Y. Qing , H. Xu , Z. Gao , W. Li , Y. Wu , ACS Appl. Energy Mater. 2020, 3, 2996.

[34] R. K. Singh , A. Schechter , Electrochim. Acta 2018, 278, 405

[35] Y. Ding , Y. Li , Y. Xue , B. Miao , S. Li , Y. Jiang , X. Liu , Y. Chen , Nanoscale 2019, 11, 1058. 30569934 10.1039/c8nr08104b

[36] Q. He , Y. Wan , H. Jiang , Z. Pan , C. Wu , M. Wang , X. Wu , B. Ye , P. M. Ajayan , L. Song , ACS Energy Lett. 2018, 3, 1373.

[37] L. Yu , S. Sun , H. Li , Z. J. Xu , Fundamental Research 2021, 1, 448.

[38] S. Haghverdi Khamene , C. van Helvoirt , M. N. Tsampas , M. Creatore , J. Phys. Chem. C. 2023, 127, 22570. 10.1021/acs.jpcc.3c05002 PMC10683065 38037639

[39] X. Gao , X. Bai , P. Wang , Y. Jiao , K. Davey , Y. Zheng , S.‐Z. Qiao , Nat. Commun. 2023, 14, 5842. 37730706 10.1038/s41467-023-41588-w PMC10511637

[40] B. Malik , K. Vijaya Sankar , S. K. T. Aziz , S. Majumder , Y. Tsur , G. D. Nessim , J. Phys. Chem. C. 2021, 125, 23126.

[41] M. Zeng , J. Wu , Z. Li , H. Wu , J. Wang , H. Wang , L. He , X. Yang , ACS Sustainable Chem. Eng. 2019, 7, 4777.

[42] X. Liu , H. Qin , Z. Ye , D. Yao , W. Miao , S. Mao , ACS ES&T Engineering 2022, 2, 853.

[43] G. Gao , M. Pan , C. D. Vecitis , J. Mater. Chem. A. 2015, 3, 7575.

[44] B. S. Yeo , A. T. Bell , J. Phys. Chem. C. 2012, 116, 8394.

[45] A. Sohel , M. S. N. Kovilakath , P. J. Gogoi , H. Ansari , P. Phukan , S. Bag , N. S. John , A. Baksi , Small 2024, 20, 2405160. 10.1002/smll.202405160 39109948

[46] N. N. Rao , C. Alex , M. Mukherjee , S. Roy , A. Tayal , A. Datta , N. S. John , ACS Catal. 2024, 14, 981.

[47] N. R. S. Farley , S. J. Gurman , A. R. Hillman , Electrochim. Acta 2001, 46, 3119

[48] R. Lin , L. Kang , T. Zhao , J. Feng , V. Celorrio , G. Zhang , G. Cibin , A. Kucernak , D. J. L. Brett , F. Corà , I. P. Parkin , G. He , Energy Environ. Sci. 2022, 15, 2386.

[49] Z. G. Wu , M. Munoz , O. Montero , Adv. Powder Technol. 2010, 21, 165.

[50] N. V. Tverdova , O. A. Pimenov , G. V. Girichev , S. A. Shlykov , N. I. Giricheva , V. E. Mayzlish , O. I. Koifman , J. Mol. Struct. 2012, 1023, 227.

[51] S. Chenakin , N. Kruse , J. Phys. Chem. C. 2019, 123, 30926.

[52] Y.‐H. Kwon , X. Yang , Z. Wu , Z. Fan , W. Xu , C.‐M. Jon , J. Phys. Chem. C. 2022, 126, 12492.

[53] V. Vedharathinam , G. G. Botte , Electrochim. Acta 2013, 108, 660

[54] D. Chen , X. Xiong , B. Zhao , M. A. Mahmoud , M. A. El‐Sayed , M. Liu , Adv. Sci. 2015, 3, 1500433. 10.1002/advs.201500433 PMC5067601 27812474

[55] D. Wang , J. Zhou , Y. Hu , J. Yang , N. Han , Y. Li , T.‐K. Sham , J. Phys. Chem. C 2015, 119, 19573.

[56] H. Dau , P. Liebisch , M. Haumann , Anal. Bioanal. Chem. 2003, 376, 562. 12802563 10.1007/s00216-003-1982-2

[57] F. T. Haase , A. Bergmann , T. E. Jones , J. Timoshenko , A. Herzog , H. S. Jeon , C. Rettenmaier , B. R. Cuenya , Nat. Energy 2022, 7, 765.

[58] X. Su , Y. Wang , J. Zhou , S. Gu , J. Li , S. Zhang , J. Am. Chem. Soc. 2018, 140, 11286. 30111100 10.1021/jacs.8b05294

[59] M. Schalenbach , A. R. Zeradjanin , O. Kasian , S. Cherevko , K. J. J. Mayrhofer , Int. J. Electrochem. Sci. 2018, 13, 1173

[60] K. Dharmaraj , R. Hanna , I. Lauermann , R. Bagacki , F. Xi , E. Kemppainen , R. Schlatmann , S. Calnan , ACS Sustainable Chem. Eng. 2024, 12, 9908.

[61] L. Xiao , C. Cheng , T. Yang , J. Zhang , Y. Han , C. Han , W. Lv , H. Tan , X. Zhao , P. Yin , C. Dong , H. Liu , X. Du , J. Yang , Adv. Mater. 2024, 36, 2411134. 10.1002/adma.202411134 39279569

[62] X. Kang , F. Yang , Z. Zhang , H. Liu , S. Ge , S. Hu , S. Li , Y. Luo , Q. Yu , Z. Liu , Q. Wang , W. Ren , C. Sun , H.‐M. Cheng , B. Liu , Nat. Commun. 2023, 14, 3607. 37330593 10.1038/s41467-023-39386-5 PMC10276855

[63] H. Liu , R. Xie , Y. Luo , Z. Cui , Q. Yu , Z. Gao , Z. Zhang , F. Yang , X. Kang , S. Ge , S. Li , X. Gao , G. Chai , L. Liu , L. Liu , B. Liu , Nat. Commun. 2022, 13, 6382. 36289229 10.1038/s41467-022-34121-y PMC9605970

[64] Y. Luo , L. Tang , U. Khan , Q. Yu , H.‐M. Cheng , X. Zou , B. Liu , Nat. Commun. 2019, 10, 269. 30655511 10.1038/s41467-018-07792-9 PMC6336864

[65] M. A. Peck , M. A. Langell , Chem. Mater. 2012, 24, 4483.

[66] E. Gioria , S. Li , A. Mazheika , R. Naumann d'Alnoncourt , A. Thomas , F. Rosowski , Angew. Chem., Int. Ed. 2023, 62, 202217888. 10.1002/anie.202217888 36999638

[67] L. Wang , X. Ge , Y. Li , J. Liu , L. Huang , L. Feng , Y. Wang , J. Mater. Chem. A. 2017, 5, 4331.

[68] Y. Li , X. Zhang , A. Hu , M. Li , Int. J. Hydrogen Energy 2018, 43, 22012.

[69] M. S. Naduvil Kovilakath , C. Alex , N. N. Rao , D. Bagchi , A. Tayal , S. Peter , N. S. John , Chem. Mater. 2024, 36, 5343.

[70] W. A. Caliebe , V. Murzin , A. Kalinko , M. Görlitz , AIP Conf. Proc. 2019, 2054, 060031

[71] F. Yang , M. J. Kim , M. Brown , B. J. Wiley , Adv. Energy Mater. 2020, 10, 2001174.

