# Supplementary material for: Accelerated Electro‐Conversion of a Nickel Coordination Complex for Hybrid Water Electrolysis
Source: Small. 2025 Aug 29;21(41):e07907. doi: 10.1002/smll.202507907 (PMC12530016; doi:10.1002/smll.202507907)
Supplement: Supplementary file 1 — Supporting Information [file SMLL-21-e07907-s002.docx]

**Supporting information**

**Accelerated Electro-conversion of a Nickel Coordination Complex for Hybrid Water Electrolysis**

Nikhil N. Rao,^a,b^ Avani Anil Kumar,^a^ Peter Kúš,^c^* Chandraraj Alex,^d^ Muhammed Safeer N. K.,^a^ Tomáš Hrbek,^c^ Iva Matolínová,^c^ Neena S. John^a^*

^a^*Centre for Nano and Soft Matter Sciences, Shivanapura, Bengaluru 562162, India.*

^b^*Manipal Academy of Higher Education, Manipal 576104, India.*

*^c^Charles University, Faculty of Mathematics and Physics, Department of Surface and Plasma Science, V Holešovičkách 2, 180 00, Prague 8, Czech Republic.*

^d^*Institute of Inorganic Chemistry, Kiel University, Otto-Hahn-Platz 10, 24118 Kiel, Germany*

*Corresponding author Email: [peter.kus@mff.cuni.cz](mailto:peter.kus@mff.cuni.cz); [jsneena@cens.res.in](mailto:jsneena@cens.res.in)

1. **Characterization of NiHyd@CNT**

Figure S1. (a) Powder XRD pattern of NiHyd@CNT-PC, (b) FTIR spectra of NiHyd, FESEM micrograph of (c) NiHyd, and (d) NiHyd@CNT-PC, (e) TEM micrograph of NiHyd@CNT-PC.

Table S1. FTIR stretching frequencies and corresponding bond assignments for NiHyd@CNT and Activated NiHyd@CNT.

| Stretching Frequency (cm⁻¹) | Bond Assignment | Reference |
| --- | --- | --- |
| NiHyd@CNT-PC | | |
| 3440 | O–H stretching (ethanol traces) | ^1^ |
| 3293 | Symmetric N–H_2_ stretching | ^2^ |
| 3234 | Asymmetric N–H_2_ stretching |  |
| 1608 | N–H_2_ bending | ^2–4^ |
| 1574 | N–H_2_ bending |  |
| 1173 | N–H_2_ twisting |  |
| 970 | N–N stretching of bidentate N_2_H_4_ ligand |  |
| 647 | NH_3_ rocking vibration (Ni(NH_3_)_6_ Cl_2_ impurity) | ^4,5^ |
| 611 | NH_3_ symmetric distortion (Ni(NH_3_)_6_Cl_2_ impurity) |  |
| NiHyd@CNT | | |
| 3650 | Fundamental O–H stretching | ^6,7^ |
| 3443 | H-bonded O–H stretching |  |
| 1635 | H–O–H bending (adsorbed or structural water) | ^8^ |
| 1386 | O–H bending (lattice OH) |  |
| 1239 | C–F stretching in CF_2_ groups (Nafion binder) | ^9^ |
| 1064 | SO_3_ vibrations (dissociated sulfonic group from Nafion binder) |  |
| 540 | In-plane O–H deformation | ^10^ |
| 454 | Ni–O stretching vibration |  |

1. **Electrochemical studies**

Figure S2. Cyclic voltammograms of (a) Ni felt in 0.1 M hydrazine + 1 M KOH, (b) β-Ni(OH)_2_@CNT in 1 M KOH during activation, (c) α-Ni(OH)_2_ in 1 M KOH during activation, (d) Activated catalysts of NiHyd@CNT, β-Ni(OH)_2_@CNT, and α-Ni(OH)_2_ in 0.33 M urea + 1 M KOH with the potentials converted into RHE scale, (e) NiHyd grown on various carbon additives, rGO, VC, CNT, and pristine complex in 0.20 M urea + 1 M KOH, (f) Activated NiHyd@CNT with different amounts of CNT in 0.20 M urea + 1 M KOH, (g) NiHyd@CNT with different catalyst mass loading in 0.20 M urea + 1 M KOH, and (h) NiHyd@CNT and NiOOH@CNT in 0.20 M urea + 1 M KOH.

Figure S3. Electrochemical activation CVs recorded (a) at different potential windows, (b) at different scan rates; CVs recorded in 0.33 M urea + 1 M KOH at a scan rate of 10 mV/s (c) for NiHyd@CNT activated under different potential windows, (d) for NiHyd@CNT activated under different scan rates

**Note:** To explore the influence of the activation potential window, activation was performed in 1 M KOH across several ranges starting just beyond the Ni²⁺/Ni³⁺ redox onset: 0–0.50 V, 0–0.60 V, 0–0.70 V, and 0–0.80 V vs. Hg/HgO (Figure S3(a)). UOR activity progressively improved from the 0–0.50 V to 0–0.70 V activation, with the best performance at 0–0.70 V (Figure S3(c)), followed by a slight decrease at 0–0.80 V. Overall, all activated samples showed comparable UOR performance, suggesting that beyond a certain threshold, increasing the potential window offers minimal additional benefit. This is likely because the key activation steps—hydrazine ligand removal (below ~0.40 V) and Ni^2+^ → Ni^3+^ oxidation (0.45–0.60 V vs. Hg/HgO)—occur within all tested potential ranges.

To determine an optimal scan rate for electrochemical activation, we referred to Creatore et al.,^11^ who showed that low scan rates (e.g., 10 mV/s) promote Ni^3+^ accumulation, beneficial for catalytic processes such as UOR. We systematically tested various rates (Figure S3(b)), and observed that activation at 10 mV/s yielded the highest UOR activity (Figure S3(d)). At faster scan rates (50–100 mV/s), reduced anodic dwell time limited Ni^2+^ → Ni^3+^ oxidation, while very slow rates (e.g., 5 mV/s) prolonged the cathodic dwell time at 0 V vs. Hg/HgO, favoring Ni^3+^ reduction to Ni^2+^. Thus, 10 mV/s likely offers the optimal balance—providing sufficient anodic dwell time for Ni^3+^ formation without excessive cathodic reduction—resulting in more effective catalyst reconstruction and enhanced UOR performance.

Figure S4. (a) Step voltage amperometric test recorded in 1 M KOH and 0.33 M urea + 1 M KOH, (b) LSVs recorded in 1 M KOH and 0.33 M urea + 1 M KOH using RRDE, (c) Zoomed figure of the recorded LSVs

**Note:** In the step voltage method, chronoamperometry was performed at 50 mV intervals from 0.45 to 0.70 V vs. Hg/HgO for 60 s at each step, in both 1 M KOH (blank) and 0.33 M urea + 1 M KOH electrolytes, under rotation (1600 rpm, Figure S4(a)). The current efficiency (in %) towards UOR is calculated by subtracting the current in the absence of urea from that in the presence of urea at a given potential and dividing by the total current obtained in the presence of urea. The current efficiency towards UOR in the potential range 0.45 V - 0.70 V vs. Hg/HgO ranges between 85.6 and 99.2%, which highlights the excellent selectivity of the catalyst towards UOR (Figure 1(i) in main text).

To further confirm this, RRDE experiments were carried out in both electrolytes under identical rotation speeds (1600 rpm), with the Pt ring electrode held at −0.50 V vs. Hg/HgO to selectively detect oxygen via the oxygen reduction reaction (ORR). As shown in Figure S4(b & c)), the ring current increases in 1 M KOH starting around 0.55 V vs. Hg/HgO, consistent with the onset of OER. In contrast, a strong suppression of ring current in urea-containing electrolyte confirms effective suppression of OER.

Figure S5. CV recorded in 0.33 M urea + 1 M KOH at a scan rate of 10 mV/s

1. **Calculation of turnover frequency (TOF)**

$$\mathbf{TOF=}\frac{\boldsymbol{j}\boldsymbol{\times}\boldsymbol{A}}{\boldsymbol{x}\boldsymbol{\times}\boldsymbol{n}\boldsymbol{\times}\boldsymbol{F}}$$

*A* is the area of working electrode (cm^2^), *x* is the number of electrons transferred in UOR (i.e. 6) and *F* is the Faraday’s constant (96,485 C mol^−1^).

Table S2. Calculation of TOF

| Catalyst | Area under oxidation peak  ($\boldsymbol{\times}\boldsymbol{10}^{\boldsymbol{-4}}$AV) | Charge corresponding to NiOOH formation= Area under the oxidation peak/scan rate  (C) | Number of NiOOH species formed (n) = Charge/(No. of electrons involved$\boldsymbol{\times}$ Faraday’s constant)  (nmol) | Current density (*j*) at 0.60 V vs. Hg/HgO (mA/cm^2^) | TOF at 0.60 V vs. Hg/HgO (in s^-1^) |
| --- | --- | --- | --- | --- | --- |
| NiHyd@CNT | 1.76 | 0.01763 | 182.7 | 110 | 0.0728 |
| β-Ni(OH)_2_@CNT | 2.52 | 0.02525 | 261.7 | 42.2 | 0.0195 |
| α-Ni(OH)_2_@CNT | 1.36 | 0.01357 | 140.6 | 23.4 | 0.0201 |

1. **Comparison of the work with catalysts reported in literature**

Table S3. Comparison of the present study with reported UOR catalysts

| Catalyst | UOR onset  (V vs. RHE) | Tafel slope  (mV/dec) | AEM electrolyser demonstrated | Potential vs. RHE required to achieve 100 mA/cm^2^ | Reference |
| --- | --- | --- | --- | --- | --- |
| NiHyd@CNT pre-catalyst on GCE | 1.35 | 21.6 | Yes | 1.503 | This work |
| NiCo LDH on Ni foam | 1.2 | - | No | > 1.6 | ^12^ |
| NiS/Ni_2_S_3_-Ni@NCNT on Ni foam | 1.32 | 39 |  | > 1.6 | ^13^ |
| MoO_2_/NiMOF/eGO on carbon cloth | ~1.3 | 31 |  | > 1.5 | ^14^ |
| Ni_3_N/Ni_3_S_2_ on GCE | > 1.35 | 30.2 |  | > 1.5 | ^15^ |
| Se-doped Ni(OH)_2_ on GCE | ~1.3 | 67.4 |  | ~1.5 | ^16^ |
| CoNi@N-doped CNT on GCE | ~1.35 | 58.1 |  | ~1.5 | ^17^ |
| Ni phosphide pre-catalyst on GCE | 1.33 | - |  | > 1.5 | ^18^ |
| FeCoNiF_2_ on carbon cloth | 1.36 | - |  | ~1.6 | ^19^ |
| NdNiO_3_-NiO on GCE | 1.35 | 41.7 |  | >1.6 | ^20^ |

1. **Electrochemical studies – Double-layer capacitance and impedance studies**

Figure S6. CVs of (a) NiHyd@CNT, (b) β-Ni(OH)_2_@CNT, (c) α-Ni(OH)_2_@CNT recorded in non-faradaic potential window at various scan rates, Nyquist plots of (d) NiHyd@CNT, (e) β-Ni(OH)_2_@CNT, (f) α-Ni(OH)_2_@CNT recorded in 0.33 M urea + 1 M KOH fitted with equivalent circuit (inset).

Table S4. Impedance fitting parameters.

| Catalyst | Potential (V vs. Hg/HgO) | Rs (Ω) | R_1_ (Ω) | R_2_ (Ω) | CPE2-T (F) | CPE2-P (F) | CPE1-T  (F) | CPE1-P (F) | χ^2^ |
| --- | --- | --- | --- | --- | --- | --- | --- | --- | --- |
| **NiHyd@CNT** | 0.46 | 11.7 | 14.0 | 37 | 0.00221 | 0.7348 | 0.000846 | 0.441 | 0.000621 |
|  | 0.50 | 12.2 | 10.0 | 19.4 | 0.00358 | 0.7264 | 0.000757 | 0.455 | 0.000781 |
| **β-Ni(OH)_2_@CNT** | 0.46 | 10.5 | 16.2 | 216.2 | 0.000292 | 0.6748 | 0.000550 | 0.436 | 0.000196 |
|  | 0.50 | 11.4 | 23.3 | 91.9 | 0.00275 | 0.4818 | 0.000575 | 0.421 | 0.000423 |
| **α-Ni(OH)_2_@CNT** | 0.46 | 11.0 | 59.5 | 235.9 | 0.00189 | 0.3851 | 0.0000865 | 0.691 | 0.000159 |
|  | 0.50 | 12.6 | 65.2 | 143.9 | 0.00637 | 0.5001 | 0.000101 | 0.667 | 0.000286 |

**Note:** The noise-free impedance data points at 0.46 V and 0.50 V vs. Hg/HgO are fitted (Figures S3(d-f)) with the typical UOR equivalent circuit shown in the inset of Figure S3(f) and the fitting parameters are tabulated in Table S4. The components of the UOR equivalent circuit include - solution resistance (Rs), faradaic resistance R_1_ and CPE1 (constant phase element) corresponding to the indirect mechanism of UOR, and faradaic resistance - R_2_ and constant phase element - CPE2 corresponding to the direct mechanism of UOR.^21^

Figure S7. (a) Nyquist plot recorded in 0.33 M urea + 1 M KOH at a scan rate of 10 mV/s, (b) Zoomed inset

Table S5. Impedance fitting parameters.

| Catalyst | Potential (V vs. Hg/HgO) | Rs (Ω) | R_1_ (Ω) | R_2_ (Ω) | CPE2-T (µF) | CPE2-P (F) | CPE1-T  (µF) | CPE1-P (F) | χ^2^ |
| --- | --- | --- | --- | --- | --- | --- | --- | --- | --- |
| **NiHyd** | 0.50 | 12.0 | 20.1 | 185.8 | 2 | 0.9595 | 645 | 0.339 | 0.000718 |
| NiHyd@CNT | 0.50 | 12.2 | 10.0 | 19.4 | 3580 | 0.7264 | 757 | 0.455 | 0.000781 |
| NiHyd@CNT post-stab | 0.50 | 5.0 | 13.8 | 29.4 | 7350 | 0.5463 | 0.421 | 0.849 | 0.00436 |

1. **Catalyst characterization post-activation and post-stability study**

Figure S8. (a) Powder XRD pattern of NiHyd@CNT, (b) Ni K-edge XANES plots of NiHyd@CNT-PC and NiHyd@CNT, (c) R-space FT-EXAFS plots of pristine, activated, and post-stability sample of NiHyd@CNT-PC, Fitted FT-EXAFS plots of (d) NiHyd@CNT-PC, (e) NiHyd@CNT, and (f) NiHyd@CNT after the amperometric stability test.

Table S6. EXAFS Fitting parameters

| Sample | Bond | R (Å) | CN | ΔE (eV) | σ^2^ (Å^2^) | R-factor (%) | k-range | R-range (Å) |
| --- | --- | --- | --- | --- | --- | --- | --- | --- |
| NiHyd@CNT-PC | Ni-N_1_  Ni-N_2_ | 2.052 ± 0.005  2.253 ± 0.007 | 2.00 ± 0.11  4.00 ± 0.10 | 2.78 ± 0.24 | 0.0014 ± 0.0005  0.0075 ± 0.0005 | 0.098 | 3 - 10 | 1.1 - 2.4 |
| NiHyd@CNT | Ni-O  Ni-Ni | 2.035 ± 0.010  3.091 ± 0.012 | 5.84 ± 0.27  4.07 ± 0.45 | 2.50 ± 0.99 | 0.0111 ± 0.0012  0.0128 ± 0.0015 | 0.87 | 3 – 9 | 1 - 3.6 |
| Post-stability NiHyd@CNT | Ni-O  Ni-Ni | 2.053 ± 0.008  3.087 ± 0.010 | 5.94 ± 0.26  5.74 ± 0.52 | 1.28 ± 0.82 | 0.0075 ± 0.0007  0.0109 ± 0.0009 | 0.66 | 3 – 9 | 1 - 3.6 |

Note: S_0_^2^ is amplitude reduction factor fixed as 0.787 (From Ni foil data), N is coordination number, R is bond length between absorber (Ni) and scatter (O, N, & Ni), σ^2^ is mean squared disordered term, ∆E is energy shift, R factor extracted from EXAFS fitting of the coordination shells.

1. ***In situ* Raman and X-ray absorption studies**


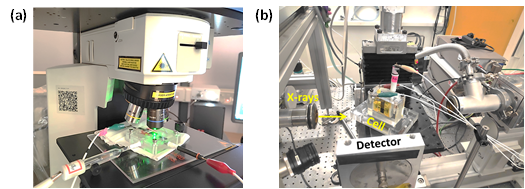


Figure S9. (a) Optical image of (a) in situ Raman setup; (b) of in situ XAS setup

Figure S10. (a) In situ Ni K-edge XANES spectra of catalysts and control samples at various potentials and XANES of standards, Magnified view of Ni K-edge XANES plots of (b) NiHyd@CNT, and (c) β-Ni(OH)_2_@CNT

Table S7. Edge position and oxidation state of Ni calculated from in situ XANES data

| Sample | Edge Position (eV) | Oxidation state |
| --- | --- | --- |
| NiHyd@CNT (OCP, 1 M KOH) | 8343.75 | 2.42 |
| NiHyd@CNT (0.54 V, 1 M KOH) | 8344.64 | 2.71 |
| NiHyd@CNT (0.54 V, 0.33 M U + 1 M KOH) | 8344.32 | 2.61 |
| β-Ni(OH)_2_@CNT (OCP, 1 M KOH) | 8343.46 | 2.33 |
| β-Ni(OH)_2_@CNT (0.54 V, 1 M KOH) | 8343.72 | 2.42 |
| β-Ni(OH)_2_@CNT (0.54 V, 0.33 M U + 1 M KOH) | 8343.74 | 2.42 |
| Standard NiO | 8342.52 | 2.00 |
| Standard LaNiO_3_ | 8345.61 | 3.00 |

1. **AEM electrolyser studies**

Figure S11. I-V curves acquired for various catalysts in AEM electrolyser assembly (a) NiHyd@CNT (on CFP)||Pt/C in 1 M KOH and 0.33 M urea + 1 M KOH (without iR-correction), (b) NiHyd@CNT(on CFP) or RuO_2_ or Ni(OH)_2_@CNT ||Pt/C in 0.33 M urea + 1 M KOH (without iR-correction), (c) NiHyd@CNT (on CFP)||Pt/C at different operating temperatures (without iR correction), (d) NiHyd@CNT (on Ni felt) or Ni(OH)_2_@CNT (on Ni felt) or bare Ni felt||Pt/C in 0.33 M urea + 1 M KOH (iR-corrected), (e) NiHyd@CNT (on Ni felt)||Pt/C in 0.33 M urea + 1 M KOH before and after the stability test (iR-corrected), (f) Potentiostatic EIS of NiHyd@CNT (on Ni felt)||Pt/C recorded in 0.33 M urea + 1 M KOH at 1.6 V, before and after the stability test

**Calculation of AEM electrolyser efficiency:**^22,23^

At a current density of 0.5 A/cm^2^ using NiHyd@CNT on Ni felt as the anode and Pt/C as the cathode, the AEM electrolyser efficiency is calculated as follows:

H_2_ production rate at 0.5 A.cm^–2^ = $\frac{0.5 A.{cm}^{-2}\times1e^{-}\times1 H_{2}}{1.602\times{10}^{-19}C\times2e^{-}\times6.023\times{10}^{23}{mol}^{-1}}=2.59\times{10}^{-6} molH_{2}.{cm}^{2}.s^{-1}$

Lower Heating Value of H_2_ = $120 kJ.g^{-1}H_{2}$ =$2.42\times{10}^{5}J.{mol}^{-1}$ H_2_

H_2_ power out = $2.59\times{10}^{-6} molH_{2}.{cm}^{2}.s^{-1}\times2.42\times{10}^{5}J.{mol}^{-1}$ H_2_ = 0.627 W.cm^-2^

Electrolyser power at 0.5 A.cm^–2^ = $(0.5 A.{cm}^{-2}) ( 1.78 V)$ = 0.89 W.cm^-2^

Efficiency of AEM electrolyser = $\frac{(H_{2} power out)}{(Electrolyzer power)}$= $\frac{0.627 W.{cm}^{-2}}{0.89 W.{cm}^{-2}}$ = 70.4%

1. **Post-stability characterization**

Figure S12. Post stability characterization of NiHyd@CNT (a) FESEM micrograph, (b) and (c) HRTEM micrographs, Deconvoluted high-resolution XPS (d) Ni-2p, (e) O-1s spectra

**References:**

1. H. D. Lutz, W. Eckers, H. Haeuseler, “OH Stretching Frequencies of Solid Hydroxides and of Free OH− Ions,” *Journal of Molecular Structure* 80 (C) (1982): 221–224. <https://doi.org/10.1016/0022-2860(82)87236-0>
2. K. C. Patil, C. Nesamani, V. R. P. Verneker, “Synthesis and Characterisation of Metal Hydrazine Nitrate, Azide and Perchlorate Complexes,” *Synthesis and Reactivity in Inorganic and Metal-Organic Chemistry* 12 (4) (1982): 383–395. <https://doi.org/10.1080/00945718208063122>
3. D. Nicholls, R. Swindells, “Hydrazine Complexes of Nickel(II) Chloride,” *Journal of Inorganic and Nuclear Chemistry* 30 (8) (1968): 2211–2217. <https://doi.org/10.1016/0022-1902(68)80219-2>
4. J. W. Park, E. H. Chae, S. H. Kim, J. H. Lee, J. W. Kim, S. M. Yoon, J.-Y. Choi, “Preparation of Fine Ni Powders from Nickel Hydrazine Complex,” *Materials Chemistry and Physics* 97 (2–3) (2006): 371–378. <https://doi.org/10.1016/j.matchemphys.2005.08.028>
5. K. H. Schmidt, A. Müller, “Vibrational Spectra and Force Constants of Pure Ammine Complexes,” *Coordination Chemistry Reviews* 19 (1) (1976): 41–97. <https://doi.org/10.1016/S0010-8545(00)80404-X>
6. F. P. Kober, “Infrared Spectroscopic Investigation of Charged Nickel Hydroxide Electrodes,” *Journal of the Electrochemical Society* 114 (3) (1967): 215. <https://doi.org/10.1149/1.2426549/XML>
7. M. Casas-Cabanas, M. D. Radin, J. Kim, C. P. Grey, A. Van Der Ven, M. R. Palacín, “The Nickel Battery Positive Electrode Revisited: Stability and Structure of the β-NiOOH Phase,” *Journal of Materials Chemistry A* 6 (39) (2018): 19256–19265. <https://doi.org/10.1039/C8TA07460G>
8. D. S. Hall, D. J. Lockwood, S. Poirier, C. Bock, B. R. MacDougall, “Raman and Infrared Spectroscopy of α and β Phases of Thin Nickel Hydroxide Films Electrochemically Formed on Nickel,” *Journal of Physical Chemistry A* 116 (25) (2012): 6771–6784. <https://doi.org/10.1021/jp303546r>
9. M. Danilczuk, L. Lancucki, S. Schlick, S. J. Hamrock, G. M. Haugen, “In-Depth Profiling of Degradation Processes in a Fuel Cell: 2D Spectral-Spatial FTIR Spectra of Nafion Membranes,” *ACS Macro Letters* 1 (2) (2012): 280–285. <https://doi.org/10.1021/mz200100s>
10. P. V. Kamath, G. N. Subbanna, “Electroless Nickel Hydroxide: Synthesis and Characterization,” *Journal of Applied Electrochemistry* 22 (5) (1992): 478–482. <https://doi.org/10.1007/BF01077552>
11. S. Haghverdi Khamene, C. van Helvoirt, M. N. Tsampas, M. Creatore, “Electrochemical Activation of Atomic-Layer-Deposited Nickel Oxide for Water Oxidation,” *Journal of Physical Chemistry C* 127 (46) (2023): 22570–22582. <https://doi.org/10.1021/acs.jpcc.3c05002>
12. Y. Yang, J. A. Yuwono, T. Whittaker, M. M. Ibáñez, B. Wang, C. Kim, A. Y. Borisevich, S. Chua, J. P. Prada, X. Wang, P. Autran, R. R. Unocic, L. Dai, A. Holewinski, N. M. Bedford, “Double Hydroxide Nanocatalysts for Urea Electrooxidation Engineered toward Environmentally Benign Products,” *Advanced Materials* 36 (35) (2024): 2403187. <https://doi.org/10.1002/adma.202403187>
13. X. Guo, Y. Li, Z. Xu, D. Liu, A. Kong, R. Liu, “Interface Electron Transfer Direction-Tuned Urea Electrooxidation Over Multi-Interface Nickel Sulfide Heterojunctions,” *Small* 21 (4) (2025): 2408908. <https://doi.org/10.1002/SMLL.202408908>
14. A. Acharya, K. Mandal, N. Kumari, K. Chatterjee, “Synergistic Inclusion of Reaction Activator and Reaction Accelerator to Ni-MOF Toward Extra-Ordinary Performance of Urea Oxidation Reaction,” *Small* 21 (1) (2025): 2407377. <https://doi.org/10.1002/SMLL.202407377>
15. H. Liu, P. Wang, X. Qi, A. Yin, Y. Wang, Y. Ye, J. Luo, Z. Ren, S. Yu, J. Wei, “Accumulated Charge Density at the Interface Boosts the Urea Oxidation Reaction Activity of Ni3N/Ni3S2 Heterointerface,” *Chemical Engineering Journal* 491 (2024): 152160. <https://doi.org/10.1016/J.CEJ.2024.152160>
16. S. Song, X. Huang, Y. Yang, L. Feng, R. Li, “Se Self-Doped Ni(OH)2 for an Efficient Urea Oxidation Reaction,” *Chemical Communications* 60 (78) (2024): 10906–10909. <https://doi.org/10.1039/D4CC03975K>
17. Q. Zhang, S. Ma, Y. Xie, S. Pan, Z. Miao, J. Wang, Z. Yang, “Cobalt Incorporation Promotes CO2 Desorption from Nickel Active Sites Encapsulated by Nitrogen-Doped Carbon Nanotubes in Urea-Assisted Water Electrolysis,” *Langmuir* 40 (49) (2024): 26212–26220. <https://doi.org/10.1021/acs.langmuir.4c03711>
18. F. Yang, X. Huang, R. Li, S. Wang, L. Feng, “Insight into the High Activity of Nickel Phosphide Precatalysts for Urea Oxidation,” *Journal of Physical Chemistry C* 128 (45) (2024): 19436–19444. <https://doi.org/10.1021/acs.jpcc.4c06070>
19. T. T. N. Tran, N. D. Hai, H. D. Ngo, T. B. N. Le, N. Q. Tran, “Highly Electron-Deficient Ternary Metal Fluoride Nanocages for Overall Urea-Assisted Water Electrolysis,” *ACS Applied Energy Materials* 8 (5) (2025): 3132–3144. <https://doi.org/10.1021/acsaem.4c03262>
20. N. N. Rao, C. Alex, S. Tomar, M. S. Naduvil Kovilakath, S.-C. Lee, S. Bhattacharjee, N. S. John, “Interface-Driven Electrocatalysis: Highlighting the Role of NdNiO₃-NiO Heterointerface in Urea Electro-Oxidation,” *Applied Catalysis B: Environment and Energy* 371 (2025): 125177. <https://doi.org/10.1016/j.apcatb.2025.125177>
21. I. M. A. Mohamed, P. Kanagaraj, A. S. Yasin, W. Iqbal, C. Liu, “Electrochemical Impedance Investigation of Urea Oxidation in Alkaline Media Based on Electrospun Nanofibers towards the Technology of Direct-Urea Fuel Cells,” *Journal of Alloys and Compounds* 816 (2020): 152513. <https://doi.org/10.1016/j.jallcom.2019.152513>
22. L. Xiao, C. Cheng, T. Yang, J. Zhang, Y. Han, C. Han, W. Lv, H. Tan, X. Zhao, P. Yin, C. Dong, H. Liu, X. Du, J. Yang, “A ‘Two-Pronged’ Strategy to Boost Hydrogen Evolution Kinetics on NiFe-Based (Oxy)Hydroxides via Oxygen Deficient Ni-Mo-Fe Coordinate Structures for Ultra-Stable Ampere-Level Alkaline Overall Water Splitting,” *Advanced Materials* 36 (44) (2024): 2411134. <https://doi.org/10.1002/adma.202411134>
23. X. Kang, F. Yang, Z. Zhang, H. Liu, S. Ge, S. Hu, S. Li, Y. Luo, Q. Yu, Z. Liu, Q. Wang, W. Ren, C. Sun, H.-M. Cheng, B. Liu, “A Corrosion-Resistant RuMoNi Catalyst for Efficient and Long-Lasting Seawater Oxidation and Anion Exchange Membrane Electrolyzer,” *Nature Communications* 14 (1) (2023): 3607. <https://doi.org/10.1038/s41467-023-39386-5>
